# Structural basis for nuclear import selectivity of pioneer transcription factor SOX2

**DOI:** 10.1038/s41467-020-20194-0

**Published:** 2021-01-04

**Authors:** Bikshapathi Jagga, Megan Edwards, Miriam Pagin, Kylie M. Wagstaff, David Aragão, Noelia Roman, Jeffrey D. Nanson, Shane R. Raidal, Nicole Dominado, Murray Stewart, David A. Jans, Gary R. Hime, Silvia K. Nicolis, Christopher F. Basler, Jade K. Forwood

**Affiliations:** 1 School of Biomedical Sciences, Charles Sturt University, Wagga Wagga, NSW 2678 Australia; 2 Center for Microbial Pathogenesis, Institute for Biomedical Sciences, Georgia State University, Atlanta, GA 30303 USA; 3 Department of Biotechnology and Biosciences, University of Milano-Bicocca, piazza della Scienza 2, 20126 Milano, Italy; 4 Biomedicine Discovery Institute, Monash University, Clayton, VIC 3800 Australia; 5 Diamond Light Source, Harwell Science and Innovation Campus, Didcot, OX11 0DE UK; 6 School of Chemistry and Molecular Biosciences, Institute for Molecular Bioscience and Australian Infectious Diseases Research Centre, University of Queensland, Brisbane, QLD 4072 Australia; 7 School of Animal and Veterinary Sciences, Charles Sturt University, Wagga Wagga, NSW 2678 Australia; 8 Department of Anatomy and Neuroscience, University of Melbourne, Parkville, VIC 3010 Australia; 9 MRC Laboratory of Molecular Biology, Francis Crick Ave., Cambridge Biomedical Campus, Cambridge, CB2 0QH UK

**Keywords:** Transcription factors, Nuclear transport, X-ray crystallography

## Abstract

SOX (SRY-related HMG-box) transcription factors perform critical functions in development and cell differentiation. These roles depend on precise nuclear trafficking, with mutations in the nuclear targeting regions causing developmental diseases and a range of cancers. SOX protein nuclear localization is proposed to be mediated by two nuclear localization signals (NLSs) positioned within the extremities of the DNA-binding HMG-box domain and, although mutations within either cause disease, the mechanistic basis has remained unclear. Unexpectedly, we find here that these two distantly positioned NLSs of SOX2 contribute to a contiguous interface spanning 9 of the 10 ARM domains on the nuclear import adapter IMPα3. We identify key binding determinants and show this interface is critical for neural stem cell maintenance and for *Drosophila* development. Moreover, we identify a structural basis for the preference of SOX2 binding to IMPα3. In addition to defining the structural basis for SOX protein localization, these results provide a platform for understanding how mutations and post-translational modifications within these regions may modulate nuclear localization and result in clinical disease, and also how other proteins containing multiple NLSs may bind IMPα through an extended recognition interface.

## Abstract (web summary)

The SOX2 pioneer transcription factor performs critical roles in pluripotency and self-renewal of embryonic stem cells. Here the authors show that SOX2’s two nuclear localization signal sequences form a contiguous binding interface on the nuclear import receptor importin-α3, and provide a structural basis for the preference of SOX2 binding to IMPα3.

## Introduction

**Fig. 1:**
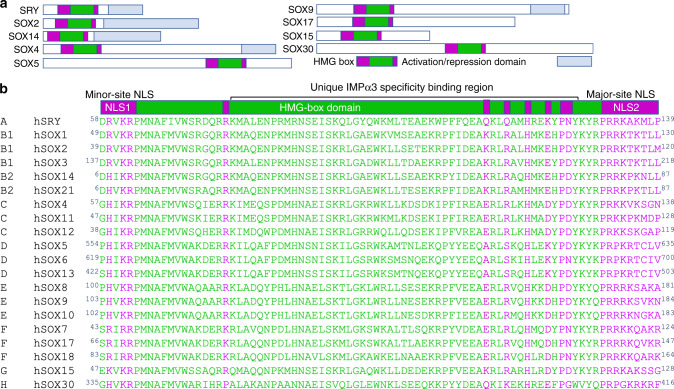
Domain organization within the SOX family. **a** The SOX family is comprised of 20 members and are grouped based on domain organization. A representative from each SOX group is shown. **b** Each SOX family member contains a highly conserved HMG domain (in green/pink), with NLSs positioned within the extremities of the HMG domain. The residues within SOX2 that form hydrogen bonds or salt bridges with IMPα3 in our crystal structure are highlighted (in pink).

The human SOX (sex-determining region Y (SRY)-related HMG-box) family of transcription factors comprises 20 members that play critical roles in organogenesis, stem cell maintenance, and cancer progression^1^. These proteins can act as both tumor suppressors or activators depending on the cellular environment^2^. SOX2 is essential for the neural development, and the self-renewal of undifferentiated embryonic and neural stem cells. It is spatially and temporally expressed during development; initial expression occurs at the preimplantation embryo stage, with restriction to the blastocyst inner cell mass and epiblast^3^. Expression is also found in the anterior ectoderm to facilitate formation of the neuroectoderm and anterior surface ectoderm^4^. During later stages of development, SOX2 expression is found in the primitive foregut endoderm^3,5^. SOX2 is one of the key factors required to convert somatic cells into induced pluripotent stem cells (PSCs), and in concert with Nanog and Oct4, plays a central role in embryonic stem cell maintenance^3,6^. These roles require precise and timely localization of SOX2 to the nucleus. To gain access to the nucleus, SOX proteins harbor two NLSs, located distally at the N- and C-terminus of the DNA-binding domain^7–15^. This arrangement is conserved across all SOX family members (Fig. 1), and mutations within these regions can impair nuclear localization, cause severe developmental disease, and are associated with poor prognosis in cancer^9,16–19^ (see also “Abstract” section).

The localization of proteins to the nucleus via the classical import pathway is an active process and requires a cargo bearing an NLS to be recognized by importin-α (IMPα)^20^. The cargo:IMPα complex is transported through the nuclear pore complex by importin-β (IMPβ)^21,22^ to the nucleus, where it is disassembled by RanGTP. Humans harbor seven IMPα isoforms (IMPα1–7), each containing an N-terminal IMPβ-binding domain (residues 1–70) and a C-terminal NLS-binding domain constructed from ten armadillo (ARM) repeats (residues 70–500). Many nuclear import cargos exhibit specificity toward these isoforms^14,23^. For example, RCC1, the Ran nucleotide exchange factor that establishes the directionality of nuclear transport, and HIV-1 integrase, responsible for integrating the HIV-1 genome into the DNA of an infected cell, bind specifically to IMPα3 (refs. ^24,25^); STAT1, a signaling molecule in the innate immune system, binds specifically to the convex C-terminal surface of IMPα5–7 (ref. ^23,26^). Ebola VP24 binds specifically to IMPα5 to selectively compete with the nuclear import of phosphorylated STAT1 (ref. ^27^). SOX proteins show remarkable isoform specificity mechanisms, and strikingly undertake isoform-specific switching during differentiation^14^. For example, neural differentiation of embryonic stem cells is mediated by IMPα isoform switching such that Oct3/4 is driven to the nucleus by IMPα1 in undifferentiated stem cells; however, during neural development, upregulation of IMPα3/5 mediates SOX2/Brn2 nuclear import and neural differentiation^14^. The molecular basis for this specificity is unclear, and understanding IMPα specificity is complicated by the seven human IMPα isoforms all containing highly conserved NLS-binding regions.

## Results

### SOX2 NLSs are bound by IMPα3 through a contiguous interface

**Fig. 2:**
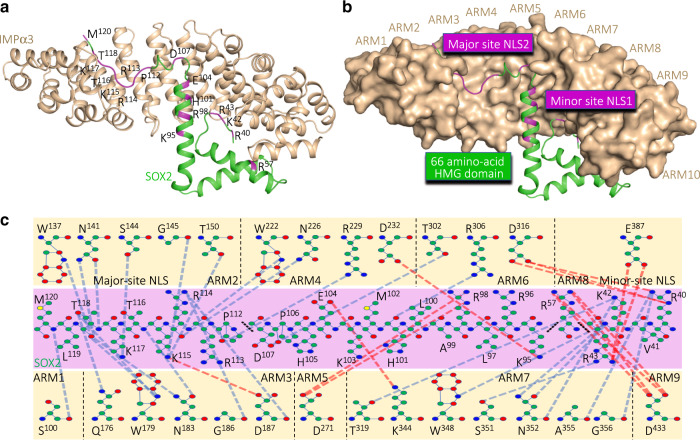
The two distally positioned NLSs of SOX2 bind to IMPα3 as part of a single continuous interface. **a** Structure of IMPα3 (shown in light brown, cartoon/ribbon format) bound to SOX2 (shown in green, cartoon/ribbon format). Residues within SOX2 that form salt bridges or hydrogen bonds with IMPα3 are highlighted pink. **b** Structural overview of SOX2 binding IMPα3 (shown in surface view), highlighting how the two distantly positioned NLSs of SOX2 are in close proximity and bind at a single interface. **c** Detailed map of the residues in the interaction interface with hydrogen bonds shown in blue and salt bridge interactions in red. Interactions are also listed in Supplementary Tables 2–4.

To better understand the mechanisms of how critical signaling regions in SOX proteins interact with nuclear import receptors to drive nuclear transport, we crystallized the HMG domain of SOX2 (comprising residues 39–127) in complex with different IMPα isoforms. The 2.3 Å resolution structure of IMPα3 bound to SOX2 (see Supplementary Table 1 for crystallographic statistics; other isoforms are discussed below), enabled the entire HMG-box domain and NLS regions of SOX2 to be reliably traced from the electron density. SOX2 bound IMPα3 through an extensive and contiguous interface across ARM domains 1–9 of IMPα3 (Fig. 2). SOX2 bound IMPα3 through an extensive and contiguous interface across ARM domains 1–9 of IMPα3 (Fig. 2). The N-terminal NLS (NLS1) was previously reported to be bipartite^7,15,28^, and therefore expected to be bound at both the major and minor sites on IMPα3. However, we found instead that, SOX2 residues Arg40, Lys42, and Arg43 were bound at the minor site (IMPα3 ARM domains 6–8; Fig. 2) and that SOX2 Arg57 was bound at ARM 9, outside of the minor site. The C-terminal NLS (NLS2) was bound in the major site of IMPα3, with SOX2 residues Pro112–Met120 bound to ARM domains 1–4 (Fig. 2). The HMG domain of SOX2, that is located between these NLS regions, formed additional interactions with IMPα3, including SOX2 Lys95 bound to ARM4; SOX2 Arg98 bound to IMPα3 ARM 5; SOX2 His101 and Asp107 bound to ARM 6; and SOX2 Glu104 bound to ARM 7 (see also Supplementary Table 2 for detailed interactions). Overall, this structure showed that, these two distally positioned NLSs within the HMG domain form a contiguous NLS interface on IMPα3 through ARM domains 1–9. That the two NLSs form a single, contiguous binding interface requires a fundamental reevaluation of how SOX proteins are recognized by the nuclear import transport machinery, how mutations in either region can cause disease, and how posttranslational modification can regulate this process^29^. More broadly, the structure provides a striking illustration of how NLS regions in different parts of a molecule can contribute to forming a contiguous NLS on the IMPα adapter.

**Fig. 3:**
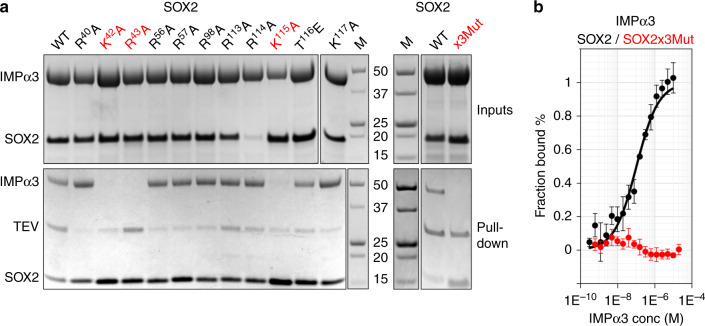
Structure-guided point mutations in SOX2 disrupt binding to IMPα3. **a** Mutations within SOX2 were introduced based on the interaction interface and were tested in pull-down assays. His-tagged SOX2 constructs was immobilized on Ni^2+^ agarose beads in the presence of IMPα3 (see input), washed, and eluted through TEV cleavage. Three single-point mutations disrupted binding: K42A, R43A, and K115A. A single SOX2 triple mutant incorporating three mutations was confirmed to not interact with IMPα3 in the equivalent pull-down experiment. See also Supplementary Fig. 6 for uncropped gels. Results were reproduced independently in three separate experiments with similar results. **b** The effect on binding was also assessed in MST assays, where SOX2 was shown to bind with 102 ± 15 nm (*n* = 3, where *n* represents three independent experiments) affinity, whereas no binding of SOX2x3Mut (red) could be detected. The data points are presented as mean values ± standard deviation.

To investigate the functional importance of the key binding determinants identified in the SOX2:IMPα3 structure, we engineered structure-guided mutations and examined their influence on nuclear localization, stem cell maintenance, and development. A total of 11 single, structure-guided mutations were assessed for their ability to disrupt interaction with IMPα3. These included R40A, K42A, R43A, R56A, R57A, R98A, R113A, R114A, K115A, T116E (to mimic phosphorylation), and K117A. We found that three of these single-point mutations reduced in vitro binding to IMPα3: K42A, R43A, and K115A (Fig. 3a). Based on a previous study demonstrating that mutating both SOX2 NLS regions impart the most dramatic reduction in nuclear localization^30^ and combining our knowledge of the structural interface, we designed a SOX2 K42A, R43A, and K115A triple mutant (SOX2x3Mut) and tested the effect on the cell biology processes that SOX2 mediates. The SOX2x3Mut binding to IMPα3 was abrogated, as shown in both pull-down (Fig. 3a) and microscale thermophoresis (MST) assays (Fig. 3b).

### IMPα3:SOX2 interface mutants affect neural stem cell biology

**Fig. 4:**
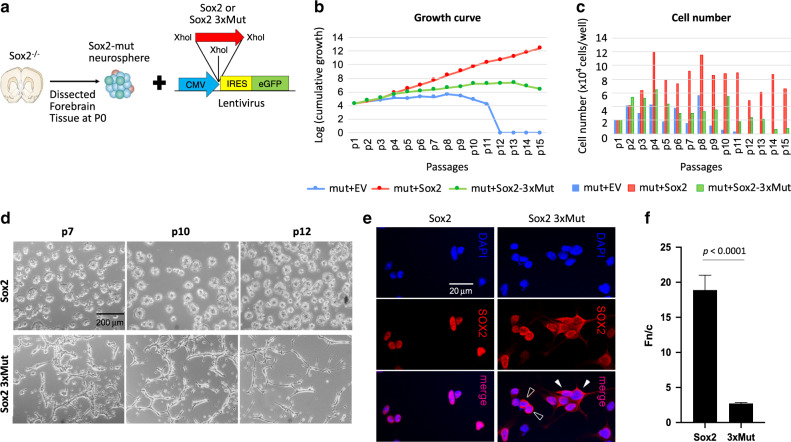
The SOX2x3Mut protein is severely impaired in its ability to maintain long-term self-renewal of SOX2-deleted NSC. **a** Schematic representation of the experimental system. **b** Cumulative growth curve of SOX2-deleted (Sox2^−/−^) NSC transduced with wild-type SOX2, or SOX2x3Mut, or empty vector (EV). **c** Number of cells recovered at each passage, after plating 20,000 cells. **d** Microscopic appearance of SOX2- and SOX2x3Mut-transduced cells, at the indicated passages. Results were reproduced independently in three separate experiments with similar results. **e** Confocal microscopy of SOX2- and SOX2x3Mut-transduced cells. Note, in SOX2x3Mut-transduced cells, a cluster of cells attaching to the slide, and possibly initiating differentiation. **f** Digitized images of SOX2 and SOX2x3Mut-transduced cells were quantitated to determine the nuclear (Fn) to cytoplasmic (Fc) florescence ratio (Fn/c) according to the formula Fn/c = (Fn-Fb)/(Fc-Fb) where Fb is background fluorescence. Results are presented as the mean Fn/c ± SEM (*n* = 46 and 96 independent cells for the SOX2 and SOX2x3Mut samples respectively). *P* value determined by using a two-tailed unpaired Welch’s *T* test in Graphpad prism 8 software; no adjustments were made for multiple comparisons. The precise *P* value of 0.0000000011 is displayed as <0.0001. See also Supplementary Fig. 7 for data point distribution used to generate mean and SEM values.

To examine the impact of these mutations on neural stem cell biology, we compared the ability of SOX2 and the SOX2x3Mut to maintain cell proliferation in a neural stem cell assay (Fig. 4). Neonatal mouse neural stem/progenitor cells (NSC) can be exponentially expanded for extended periods of time (several months) in vitro; in contrast, Sox2-deleted (Sox2^−/−^) NSC replicate slowly, and progressively lose their ability to self-renew until the culture becomes completely exhausted^31,32^. We attempted to rescue the ability of Sox2^−/−^ NSC to long-term self-renew by transducing them with lentiviruses expressing human wild-type SOX2 (ref. ^32^) or SOX2x3Mut (Fig. 4a). Sox2^−/−^ NSC transduced with wild-type Sox2 recovered the ability to efficiently self-renew (Fig. 4b, c), growing with kinetics comparable to wild-type NSC (doubling time: 41.81 ± 7.45 h for mutant cells transduced with wild-type Sox2, versus 44.22 ± 4.95 h for wild-type cells, *n* = 3 independent experiments; error is standard deviation). In contrast, NSC transduced with the SOX2x3Mut demonstrated inefficient expansion, progressively slowing until a plateau was reached, after which their numbers started to decline (Fig. 4b, c). In an independent experiment with NSC from a different Sox2 mutant mouse transduced with the same vectors, essentially identical results were obtained, with growth curves closely overlapping those of the first experiment (Supplementary Fig. 1). NSC transduced with SOX2x3Mut had a clear tendency to attach to the plastic, elongate, and aggregate (a possible sign of initial differentiation), in contrast to Sox2^−/−^ NSC transduced with wild-type SOX2, that formed neurospheres, as expected for normal cells (Fig. 4d). FACS analysis of the transduced cells compared to untransduced cells, measuring GFP expressed from the lentiviral vector, showed that in the same culture the transduced cells rapidly exceeded the untransduced cells in number, demonstrating a growth advantage over the rapidly declining SOX2^−/−^ NSC. Notably, NSC transduced with SOX2x3Mut also grew quicker and for longer than untransduced SOX2 mutant NSC (or empty-vector (EV)-transduced NSC, Supplementary Fig. 2), suggesting that SOX2x3Mut may retain some activity, though clearly too low to maintain efficient long-term growth (Fig. 4b, c). Confocal microscopy (Fig. 4e, f) demonstrated predominantly cytoplasmic localization in most of the SOX2x3Mut-transduced NSC, whether they had initiated differentiation (white arrowheads) or not (black arrowheads); in contrast, SOX2 was invariably nuclear in wild-type SOX2-transduced NSC. These results indicate a clear correlation between the predominantly cytoplasmic localization of the SOX2x3Mut, and the severe impairment of its ability to sustain long-term NSC self-renewal.

### Homologous SOX2:IMPα3 mutations affect *Drosophila* development

**Fig. 5:**
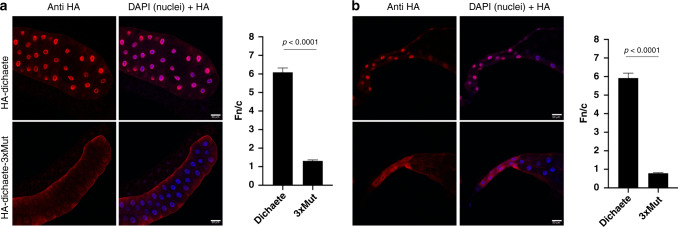
Loss of the orthologous K42, R43, and K115 residues in *Drosophila* Dichaete (SOXB 2-1) impairs nuclear localization. **a** HA-tagged Dichaete exhibits predominately nuclear localization when expressed in salivary gland cells, and **b** in salivary duct cells. In contrast, the Dichaete3xMut allele is distributed between nucleus and cytoplasm in salivary gland cells, and is strongly cytoplasmic in salivary duct cells. Scale bar = 50 µm. Digitized images were quantitated to determine the nuclear (Fn) to cytoplasmic (Fc) florescence ratio (Fn/c) as per the legend to Fig. 4, and results are presented as the mean Fn/c ± SEM (*n* = 62 and 51 (salivary gland), or 52 and 20 (salivary duct) independent cells for the HA-Dichaete and HA-Dichaete3xMut samples respectively). *P* value determined by using a two-tailed unpaired Welch’s *T* test in Graphpad prism 8 software; no adjustments were made for multiple comparisons. The precise *P* value could not be generated, but less <0.000000000000001 and displayed as <0.0001. See also Supplementary Fig. 8 for distribution of data points used to generate mean and SEM values.

The role of SOX2 in mammalian embryogenesis is well established, with Sox2^−/−^ known to be embryonic lethal in mice. The key roles of SOX2 are conserved throughout metazoans, with the SOX2 homolog in *Drosophila*, Dichaete (87% sequence identity to human SOX2 in the HMG domain and NLS-binding regions), playing a key role in central nervous system development. To examine if this interface is required for Dichaete function, we generated transgenic strains that expressed HA-tagged Dichaete or the orthologous Dichaete3xMut (Dichaete K143A, R144A, and K216 A) from an upstream activation sequence (UAS) promoter. We drove expression using ptc-Gal4 (P{GawB}ptc^559.1^) that expresses in multiple tissues during development, including third instar salivary glands. Ectopic expression of Dichaete results in developmental defects when expressed from a variety of promoters^33–35^, and we also observed that no ptc-Gal4, UAS-Dichaete adults emerged (0/63 siblings, Supplementary Fig. 3), indicating that it results in lethality when raised at 25 °C. In contrast, expression of Dichaete3xMut had no effects upon development and ptc-Gal4, UAS-Dichaete3xMut animals emerged at approximately a Mendelian ratio (37/93 siblings, Supplementary Fig. 3). We were able to isolate third instar larval salivary glands from both allelic combinations and used an anti-HA antibody to observe the intracellular localization of the ectopically expressed Dichaete proteins. Wild-type HA-Dichaete was predominantly localized in nuclei of both the polytene salivary gland cells and the salivary duct cells, whereas HA-Dichaete3xMut localization was much more cytoplasmic. This mislocalization explains why expression of the Dichaete3xMut did not produce a phenotype, as it was not efficiently translocated into the nucleus where it could have an effect upon target gene expression (Fig. 5).

### Structural basis for SOX2 specificity toward IMPα isoforms

**Fig. 6:**
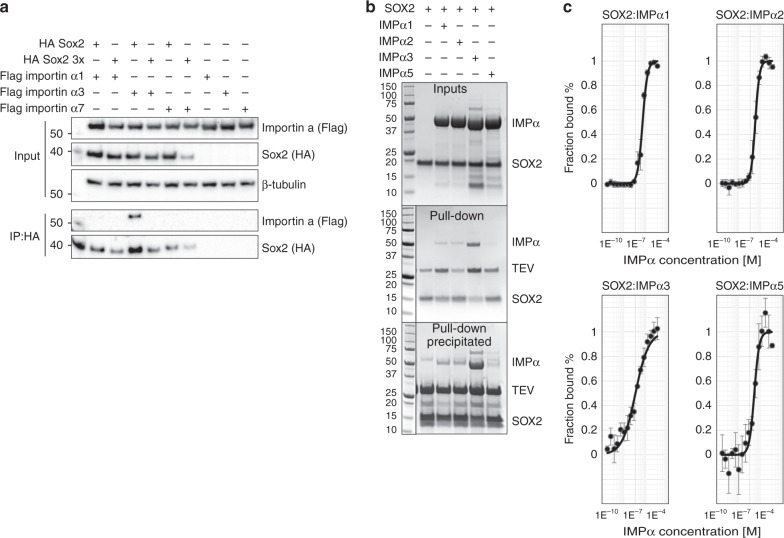
SOX2 exhibits specificity for IMPα3. **a** Co-immunoprecipitation assay performed with HA antibody on lysates of HEK293T cells expressing Flag-tagged importin from each subfamily (SF; SF1:α1, SF2:α3, SF3:α7) and HA-tagged full length SOX2 or SOX3xmut as indicated. A molecular weight marker is present in lane 1. The CoIP was repeated three times; a representative western blot is shown. Western blots were performed for HA and Flag. IP immunoprecipitation (see also Supplementary Fig. 9 for uncropped gels). **b** Pull-down assays using recombinant proteins expressed in *E. coli*. A representative from each subfamily was expressed. His-tagged SOX2 WT (wild type) was immobilized on Ni^2+^ agarose beads in the presence of SF1:α1 and α2, SF2:α3, or SF3:α5 as indicated, (see input), washed, and eluted through TEV cleavage. The elution was further concentrated by precipitation to confirm binding differences between IMPα3 and the other isoforms. See also Supplementary Fig. 10 for uncropped gels. Results were reproduced independently in three separate experiments with similar results. **c** Binding affinities of SOX2 to IMPαs within different subfamilies. Sments OX2 bound to IMPα1 with 447 ± 27 nm affinity (*n* = 3); IMPα2 with 283 ± 10 nm affinity (*n* = 3); IMPα3 with 102 ± 15 nm affinity (*n* = 3); and IMPα5 with 247 ± 30 nm affinity (*n* = 3). In each case, *n* = 3 represents three independent experiments. The data points are presented as mean values ± standard deviation. The differences in binding observed between IMPα3 and other isoforms were significant: IMPα1 *P* = 0.000042; IMPα2 *P* = 0.000064; and IMPα5 *P* = 0.0017. *P* value determined by using a two-tailed unpaired Welch’s *T* test in Graphpad prism 8 software; no adjustments were made for multiple comparisons.

SOX2 shows specificity toward IMPα isoforms during neural stem cell differentiation^14^. Moreover, many transcription factors and viral proteins exhibit specificity for IMPα isoforms, but the molecular mechanisms are unclear and analysis is complicated because of the high level of conservation of NLS-binding sites across IMPα isoforms. To assess whether SOX2 interacts differentially with IMPα isoforms, we performed an immunoprecipitation assay with representative members of each IMPα subfamily (SF; SF1:α1, SF2:α3, and SF3:α5/7; Fig. 6a). Here, we expressed Flag-tagged IMPα1, α3, and α7 together with HA-tagged SOX2, and performed HA-immunoprecipitation and western analysis. We found that SOX2 bound IMPα3, whereas interaction with IMPα1 and IMPα7 was not detected (Fig. 6a). As expected, the SOX2x3Mut demonstrated a loss of interaction with IMPα3. We performed a bead-binding assay using bacterially expressed His-tagged SOX2 immobilized on Ni^2+^ agarose that was able to bind IMPα3 directly, whereas IMPα1, α2, and α5 (same SF as IMPα7) bound more weakly (Fig. 6b). Next, we compared the affinity of these interactions using MST. Here we found that IMPα3 bound SOX2 with the strongest affinity, with a *K*_D_ of 102 ± 15 nM (mean + SD, *n* = 3), whereas IMPα1, and α5 bound with significantly lower affinity with a *K*_D_ of 447 ± 27 nM (*P* < 0.0001) and 247 ± 30 nM (*P* = 0.0017), respectively (Fig. 6c). Overall, our results indicate that SOX2 interacts most strongly with the IMPα3 isoform.

**Fig. 7:**
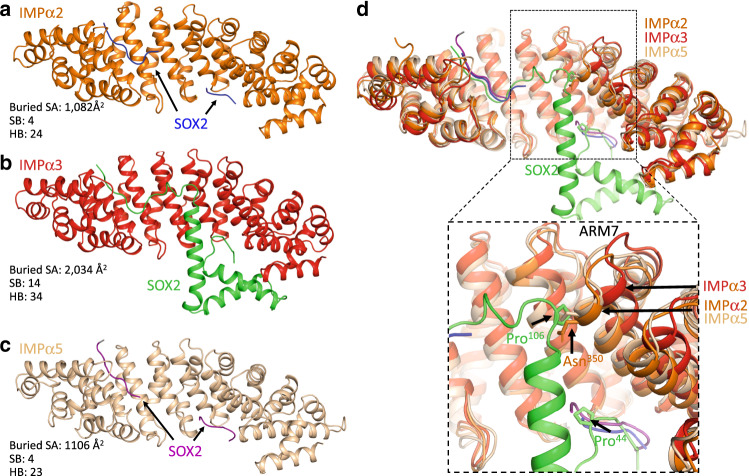
Structures of SOX2 bound to IMPα isoforms from each of the three subfamilies show significant differences in the binding interface. **a** IMPα2:SOX2 structure shown with IMPα2 (orange) and SOX2 in blue. The interface was mediated through 1082 Å^2^ of buried surface area, 4 salt bridges, and 24 hydrogen bonds. **b** The IMPα3:SOX2 structure exhibits a greater interface, with IMPα3 (red) and SOX2 in (green) burying 2034 Å^2^ of surface area, and mediated by 14 salt bridges, and 34 hydrogen bonds. **c** The IMPα5:SOX2 is depicted with IMPα5 in light brown and SOX2 in purple, buries 1106 Å^2,^ and was mediated by 4 salt bridges and 23 hydrogen bonds. **d** Superposition of the three structures highlights the positioning of ARM 7 in IMPα3 is conducive to binding the HMG domain, while IMPα2 and IMPα5 are both positioned in a conformation that prevents these interactions.

To investigate the possible basis for IMPα isoform specificity, we aligned the amino acid sequences of IMPα isoforms and compared the binding interface residues (Supplementary Fig. 4). We found that of the 28 binding interface residues on IMPα3, 25 are identical across all IMPα isoforms and the remaining 3 are conserved. This suggests that differences in the IMPα NLS-binding sites are not responsible for isoform specificity. To further probe the basis of isoform specificity, we solved the structures of SOX2 bound to both IMPα2 and IMPα5 (2.7 and 2.8 Å resolution respectively). Consistent with the cellular and in vitro binding data, we observed differences at the SOX2:IMPα isoform interfaces, with that from IMPα3 being more extensive. The IMPα3:SOX2 interface was mediated through a buried surface area of 2034 Å^2^, 14 salt bridges, and 34 hydrogen bonds. In comparison, the IMPα2:SOX2 interaction interface buried 1082 Å^2^ of surface area, and contained 4 salt bridges and 24 hydrogen bonds. The IMPα5:SOX2 interface buried 1106 Å^2^ of surface area, and contained 4 salt bridges and 23 hydrogen bonds (see Supplementary Tables 2–4 for detailed interactions). Moreover, superimposing the IMPα isoform structures indicated that local differences in IMPα structure could contribute to the differences in affinity for SOX2 (Fig. 7). Although the overall structures of the IMPα isoforms were very similar (RMSD between IMPα3 with IMPα2 and IMPα5 of 1.5 and 1.9 Å, respectively), we found that the ARM 7 domain is positioned differently in IMPα3 compared to IMPα2 and IMPα5. This region is where the SOX2 HMG domain interacts with IMPα3, but this interaction was not observed in the IMPα2 and IMPα5 crystals, where instead the HMG domain appeared to be disordered. The position adopted by the ARM 7 domain in both IMPα2 and IMPα5 would generate a steric clash with SOX2 that could impair its binding (Fig. 7). Furthermore, Pro106 of SOX2, that induces a sharp bend adjacent to the SOX-HMG domain, would clash with the main chain of both IMPα2 and α5 ARM 7. In contrast, the ARM 7 domain of IMPα3 is set back by 4 Å, and so provides a favorable interaction with the HMG domain. In addition, SOX2 cis-Pro44 is also positioned close to the ARM 7 interface. These two prolines lie at either end of the SOX2 HMG domain and facilitate the extended interactions with IMPα3 across ARM domains 1–9 that are not possible with IMPα2 and α5. The reduced binding of the R43A-SOX2 to IMPα3 was consistent with the position of ARM 7 contributing to the interaction because Arg43 forms the majority of the interactions with this region (Fig. 3). The different positioning of ARM 7 within each isoform was independent of the type of cargo bound (Supplementary Fig. 5), suggesting that this region does not adjust to accommodate different cargo.

## Discussion

SOX proteins localize to the nucleus where they alter nucleosome structure and function^36^, play critical roles in development, and are associated with many cancers. For example, mutations in the SRY protein, within either the N- or C-terminal NLS, reduce the ability of SRY to translocate to the nucleus in sex reversed patients^9,17,18^. In addition, mutations in the NLS region of SOX9 result in reduced nuclear accumulation in campomelic dysplasia patients with XY sex reversal^12^, and SOX10 mutants that fail to localize to the nucleus cause Waardenburg syndrome, resulting in sensorineural hearing defects and auditory-pigmentary disorder^37^. SOX2 plays a critical role in PSCs^3,6,14^, in neural and other stem cell type maintenance^31,32^, and in developmental and tumor biology^3–6,14,16,38–40^. SOX proteins may either maintain or antagonize tumorigenesis^38–42^. Cytoplasmic SOX9 correlates with poor clinical cancer outcomes, including both shorter disease-specific survival and relapse-free survival^19^. SOX9 is localized in the cytoplasm of 25–30% invasive ductal carcinomas and lymph node metastases, and its cytoplasmic accumulation significantly correlates with enhanced proliferation in breast tumors^43^. Cytoplasmic SOX18 correlates with poor patient outcome in adenocarcinoma and is associated with non-small cell lung cancer progression^44^. Moreover, many translational modifications occur within the SOX NLS regions (reviewed in ref. ^45^). The proposal that SOX proteins localize to the nucleus through two NLSs made it difficult to understand how mutation or modification in only one NLS would affect function because there appeared to be a high level of redundancy provided by the remaining NLS. However, our results demonstrate that these two NLS regions bind as a single continuous interface on IMPα, and so provide a more easily understood mechanistic basis for contextualizing SOX function and how it can be impaired by mutations in the NLS-HMG region of the molecule. The structural insights from our study may also assist with contextualizing how mutations in other SOX proteins may cause aberrations in nuclear transport and disease. While there are no naturally occurring mutants in the NLSs of SOX2 that have been documented, SRY mutants have been shown to impede nuclear localization and result in sex reversal. Mutations such as SRY R62G^15^, R75M^46^, and R76P^47^, located within the NLS1 (bipartite region) of SOX proteins, were shown to bind within the IMPA minor site (IMPα3 ARMs 6–8) and ARM 9 in this study. Similarly, the NLS2 region harbors mutations, such as SRY R133W^48^, shown to bind at the major site of IMPα3 (within ARM3). It is unlikely however that the interfaces identified in this study can be used to attribute all disease-causing mutations across the SOX family since these sites are also subject to complex regulation, including calmodulin binding (also shown to regulate nuclear import). This may explain for example why some disease-causing mutations, such as SRY R76P^47^ (equivalent to SOX2 Arg57), shown to be important for nuclear import regulation through calmodulin, did not disrupt the IMPA3:SOX2 interaction^8^.

**Fig. 8:**
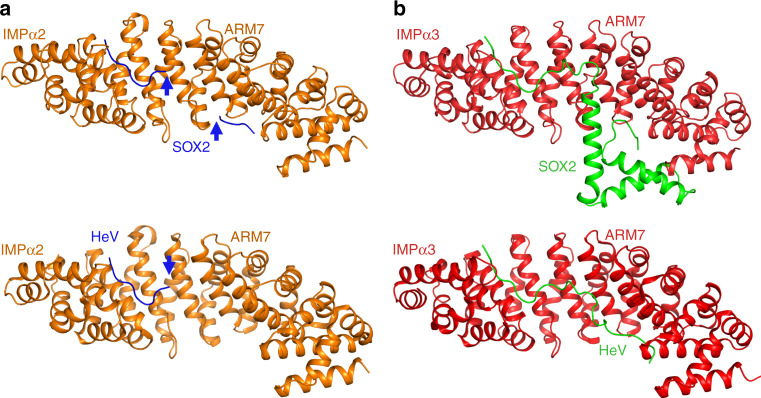
The positioning of ARM domain 7 is also important for specificity of the Hendra virus W protein. **a** Structure of IMPα2 (shown in light brown, cartoon/ribbon format) bound to SOX2 or Hendra virus W protein (HeV; shown in blue, cartoon/ribbon format). **b** Structural overview of SOX2 and HeV (shown in green, cartoon/ribbon format) bound to IMPα3 (shown in red, cartoon/ribbon format). The positioning of the ARM 7 was shown to be important for isoform specificity between IMPα2 and IMPα3.

Establishing the mechanism by which nuclear cargoes are recognized specifically by different IMPα isoforms is critical for understanding many key regulatory, developmental, and cancer-related processes. For example, neural differentiation of embryonic stem cells is mediated by IMPα isoform switching such that Oct3/4 is driven to the nucleus by IMPα1 in undifferentiated stem cells; however, during neural development, upregulation of IMPα3/5 mediates SOX2/Brn2 nuclear import and neural differentiation^14^. Moreover, SOX proteins may also use alternate pathways for import, such as IMPβ^49^, calmodulin-mediated pathway^12^, or exportin-4 (ref. ^50^), suggesting that import may be cell or tissue dependent. Structural insights into isoform specificity are limited and our present understanding of cargo binding to different receptor isoforms is limited to RCC1 (ref. ^24^), influenza A PB2 (ref. ^51^), and Henipavirus W proteins. It is noteworthy that viral cargo show some of the most remarkable specificity, and early indications suggest this may be an important strategy for viruses, since innate immune responses also require the nuclear transport of STAT1 and NF-kB through isoforms specificity of IMPα3 (ref. ^52^). Viral accessory proteins have been shown to specifically inhibit transport of these proteins to the nucleus and block innate immune responses. The ability of viruses to specifically target nuclear import to dampen immune responses, while not altering the import of cellular proteins important for cell maintenance (and viral replication), is likely an important viral replication strategy. The greater flexibility of IMPα3 compared with other IMPα isoforms is important for its binding RCC1 selectively^24,51^, whereas we have shown here that the differential positioning of ARM 7 in IMPα3 (the position of which does not change relative to ARM 6 and ARM 8 in all published IMPα3 structures—see Supplementary Fig. 5) makes an important contribution to its selective binding of SOX2, similar to that seen for the W protein of Henipaviruses^52^ (Fig. 8).

**Fig. 9:**
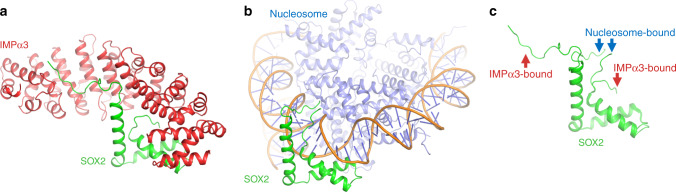
SOX2 NLS regions adopt different conformations when bound to importins or nucleosomes. **a** To gain access to the nucleus, SOX2 binds IMPα3 with the NLS regions in an extended conformation to form a single interface. **b** When localized to the nucleus, these regions adopt a closed conformation for optimal DNA binding^36^. **c** The regions are mutually exclusive and may suggest a possible release mechanism.

Finally, the NLS1 and NLS2 regions within SOX2 that mediate a single interface on IMPα3 are likely to have overlapping functions with SOX2 biology. A recent structure of SOX2 bound to nucleosomes^36^ identified that these regions may adopt strikingly different conformations (Fig. 9 and Supplementary Movie file 1). When bound to IMPα’s for nuclear import, these regions are positioned in an open conformation to allow a single continuous interface. In contrast, when bound to nucleosomes, these NLS regions are in close proximity and in a closed conformation. That the IMPα and nucleosome binding sites are overlapping suggests a possible release (and recycling) mechanism for IMPα; however, this requires further experimental investigation.

## Methods

### Protein expression

#### Plasmids for recombinant protein expression

The SOX2 HMG domain (encoding residues 39–127; UniProtKB P48431), and IMPα lacking the IMPβ-binding domain: IMPα1 (encoding residues 70–529; UniProtKB P52292), IMPα2 (encoding residues 70–529; UniProtKB P52293), IMPα3 (encoding residues 64–521; UniProtKB O00629), IMPα5 (encoding residues 73–538; UniProtKB p52294), all containing an N-terminal TEV cleavage site (ENLYFQS) were codon optimized for expression in *Escherichia coli* and synthesized by Genscript (Piscataway, NJ). The constructs were cloned into a single BamH1 site within the pET30a+ vector.

#### Recombinant expression and purification

Plasmids were transformed into BL21 (DE3) pLysS cells and expressed, as described in similar studies^53,54^, and using the following procedures. Starter cultures were inoculated into 2 L baffled flasks containing 500 mL of expression media consisting of 1% (w/v) tryptone, 0.5% (w/v) yeast extract, 0.5% glycerol, 0.05% glucose, 0.2% (w/v) α-lactose, 0.025 M NH_4_SO_4_, 0.05 M KH_2_PO4, 0.05 M Na_2_HPO4, 1 mM magnesium chloride, and 50 µg/mL kanamycin. Cells were harvested via centrifugation at 6500 × *g* and 18 °C for 30 min and resuspended in phosphate buffer (PB; 50 mM phosphate pH 8.0, 300 mM NaCl, and 20 mM imidazole). Cells were lysed using two freeze–thaw cycles, and addition of 20 mg lysozyme and 0.5 mg DNaseI.

Purification of 6xHis-tagged proteins were performed by injecting clarified cell lysate onto a GE HisTrap 5 mL column using PB, washing the column with 15 column volumes, and then eluting over 5 column volumes, using a gradient elution with high imidazole (500 mM imidazole, 300 mM NaCl, and 50 mM phosphate pH 8.0). Samples were pooled, and the affinity tag removed by TEV proteolysis. Size-exclusion purification of pooled samples was performed on a Superdex 200 pg 26/600 column, using TBS pH 8.0. Eluted proteins were pooled and concentrated using 10 kDa MW centrifuge filters. Complex formation was performed by treating the SOX2 samples with RNAse, and mixing IMPα isoforms with SOX2 in a 1:2 molar ratio. The samples were repurified using size-exclusion chromatography, concentrated using 10 kDa MW centrifuge filters, and aliquoted.

### Crystallization, data collection, and processing

All crystals were obtained using the hanging drop vapor diffusion method over a 300 μL reservoir solution. IMPα2:SOX2 was crystallized in 750 mM sodium citrate (pH 7.0) and 10 mM DTT, single rod-shaped crystals forming within 14 days. IMPα3:SOX2 crystallized in 1.2 M (NH_4_)_2_SO_4_, 0.1 M HEPES pH 6.5, with plate-shaped crystals forming within 3 days. IMPα5:SOX2 crystallized in 0.2 M sodium/potassium phosphate, 20% (w/v) PEG3350 with needle-shaped crystals forming within 7 days. X-ray diffraction data were collected at the Australian Synchrotron on the MX1 and MX2 macromolecular beam lines, using an ASDC Quantum 210r, ASDC Quantum 315r detector, and Eiger 16 M detector, respectively^55,56^. Data reduction and integration was performed using iMosflm^57^. Merging, space group assignment, scaling and selection of 5% reflections for *R*_free_ calculations were performed, using Aimless^58^ and the CCP4 suite^59^. The anisotropy of the SOX2:IMPA3 and SOX2:IMPA5 data sets was addressed, using the UCLA MBI server (https://services.mbi.ucla.edu/anisoscale/). Phasing was performed using molecular replacement in Phaser MR, with PDBID 3UL1 (ref. ^60^) used as a search model for IMPα2:SOX2, PDBID 6BW9 (ref. ^52^) for IMPα3:SOX2, PDBID 4B18 (ref. ^61^) for IMPα5:SOX2. Models were built and refined using iterative cycles of coot^62^ and maximum likelihood phenix refine^63^. The final models have been validated and deposited to the PDB with accession numbers detailed in Supplementary Table 1.

### Microscale thermophoresis

Binding affinity measurements were performed on a Monolith NT.115 (NanoTemper Technologies). Purified importins were buffer exchanged in 20 mM HEPES, 150 mM NaCl, pH 8.0, and labeled, using the Monolith his-tag labeling kit (RED-tris-NTA second generation), Nano-Temper labeling kit according to the manufacturer’s instructions. Each reaction consisted of 10 μL of the labeled SOX2 protein at 50 nM, mixed with unlabeled importins at the indicated concentrations. All experiments were measured at 25 °C with laser off/on/off times of 5/30/5 s. Experiments were conducted at 20% light-emitting diode power and 20–40% MST infrared laser power. Data from three independently performed experiments were fitted to the single binding model via the NT. Analysis software version 1.5.41 (NanoTemper Technologies) using the signal from thermophoresis + T-Jump.

### Bead-binding assays

Each assay contained 10 µM of SOX2 or SOX2 mutant immobilized on 50 µL of Ni-NTA His-bind resin (Cat#70666), and 20 µM of IMPα in a reaction volume of 300 µL. The resin was washed three times with PB (50 mM PB pH 8.0, 300 mM NaCl, and 20 mM imidazole). After the final wash, 95 µL of PB buffer and 5 µL of TEV protease (5 mg/mL) were added into each reaction, and incubated at RT for 30 min. A total of 30 µL of supernatant was removed and analyzed by SDS–PAGE. Where precipitation was used to concentrate the sample, 70 µl of the supernatant was mixed with 1 mL of 100% ethyl alcohol and incubated overnight at −20 °C. Samples were then centrifuged and the pellet dissolved in SDS–PAGE loading dye, and analyzed by SDS–PAGE.

### Primary ex vivo neural stem/progenitor cell cultures

Brain-derived NSC cultures were obtained from dissected telencephalon of two SOX2-deleted mice at postnatal day 0 (P0)^32^, and grown in fresh medium (FM): Dulbecco’s minimal essential medium (DMEM)-F12 with Glutamax (GIBCO), supplemented with 1/50 (vol/vol) B27 (Life Technologies), 1% of penicillin–streptomycin (Euroclone) supplemented with EGF (10 ng/mL, Tebu-bio) as mitogen (see also refs. ^31,32,64^).

### Lentiviral preparations

Lentiviral vectors were produced by calcium phosphate transfection into the packaging human embryonic kidney cell line 293 T, of the VSV-G plasmid (encoding ENV), CMV R8.74 (packaging), and pRSV-REV (encoding reverse transcriptase)^31,65^. Briefly, after transfection, following replacement with DMEM high glucose (Euroclone), containing 10% fetal bovine serum (FBS; Sigma), 1% penicillin–streptomycin (Euroclone), 1% of L-glutamine (Euroclone), the cells were further incubated for 48 h, and the cell supernatants were collected. Lentiviral vectors were titrated on HEK293T cells by measuring the percentage of eGFP-positive cells by flow cytometry^65^.

### NSC transduction

#### NSC transduction^31^

SOX2-deleted neurospheres were grown for two passages (3–4 days each) in FM supplemented with bFGF (10 ng/mL, Tebu-bio) and EGF (10 ng/mL, Tebu-bio), and for three more passages in FM supplemented with EGF only. For passaging, neurospheres were first incubated in 0.25% trypsin (GIBCO) for 5 min at 37 °C and, subsequently, ovomucoid (Leibovitz’s L15 medium (GIBCO) containing trypsin inhibitor (Sigma), bovine serum albumin (BSA; Sigma), and 40 µg/mL DNaseI (Sigma) for 5 min at 37 °C). Neurospheres were then carefully dissociated mechanically by gently pipetting up and down, centrifuged at 265 × *g* for 4 min and cells were resuspended in 1 mL of FM, counted, and seeded at a density of 5 × 10^5^ cells/T25 cm^2^ flask/5 mL, in FM with EGF as mitogen. After 4 h, SOX2-deleted NSCs were transduced with a GFP-SOX2-expressing lentivirus^32,66^, or with the same vector (EV) expressing GFP only (for control) or carrying SOX2x3Mut, at a multiplicity of infection of 8 for each vector, individually. Cells were incubated overnight at 37 °C. The virus was removed by medium change at 24 h: cells were centrifuged at 180 × *g* for 4 min and resuspended in FM with EGF. At every passage, every 3–4 days, cells were dissociated to single cells as above, counted, and replated in FM with EGF at a density of 20,000 cells/well/1 mL, to generate a cumulative growth curve. At every passage, aliquots of transduced cells (50,000–100,000 cells, from pooled wells) were fixed using 2% paraformaldehyde (PFA) and analyzed for GFP fluorescence by CytoFLEX (Beckman-Coulter) to determine the percentage of transduced cells: 10,000 events were analyzed for each sample.

Mice homozygous for a “floxed” Sox2 allele were crossed with mice compound heterozygotes for a βgeo gene knocked into the Sox2 gene (generating a null Sox2 mutation), and a nestin-cre transgene (which deletes the floxed Sox2 allele specifically in the nervous system), to obtain mutant mice with homozygous Sox2 deletion in the brain (Favaro et al.^32^). Control Sox2-wild-type mice are generated in the same crosses when the nestin-cre gene is not inherired, and have a Sox2 floxed allele together with an intact Sox2 gene. Mice were sacrificed at P0 to obtain forebrains for NSC cultures (sex was indifferent). The lines (Favaro et al.^32^) are maintained by matings between cousins, and outbred every two to three generations with B6D2F1 mice, to maintain the mutant alleles. Mice were housed at a temperature of 19–23 °C, with 40–60% humidity, and a 13 h light/11 h dark cycle. The experiments were approved by the Italian Ministry of Health as conforming to the relevant regulatory standards.

### Immunocytochemistry

Transduced SOX2-deleted NSCs were dissociated to single cells and seeded on Matrigel™-coated glass coverslips at a density of 80,000 cell/coverslip. After 4 h, cells were fixed for 20 min with 4% PFA in phosphate-buffered saline (PBS; pH 7.4) and rinsed three times with PBS. Coverslips were then incubated for 90 min in PBS containing 10% normal goat serum, 0.2% Triton-X100 at room temperature. Coverslips were then incubated with the primary anti-SOX2 antibody (mouse monoclonal IgG2a, 1:100, R&D Systems), overnight at 4 °C. After thorough washing with PBS, cells were incubated for 45 min at room temperature with secondary goat anti-mouse IgG2a cross-adsorbed secondary antibody, Alexa Fluor 594, (Thermo Fisher Scientific, Catalog# A-21135, 1:1000). Coverslips were rinsed three times in PBS and mounted on glass slides with Fluoromount (Sigma) with DAPI (4′,6-diamidino-2-phenylindole).

### *Drosophila* expression studies

The *Drosophila melanogaster Dichaete* coding sequence as cloned into pUASTattB with modification of the stop codon to introduce Gly–Ser residues followed by a 3xHA tag. The *Dichaete*x3Mut transgene was generated by mutating K42, R43, and K115 to alanine residues (Genscript). Both transgenes were introduced into the same genomic position in the *Drosophila* genome (BestGene, Inc.) and expression was induced at 25 °C, using the ptc-Gal4 (P{GawB}ptc^559.1^) driver (Bloomington *Drosophila* Stock Center). Immunostaining was conducted as described previously^67^, and involved salivary glands dissected from wandering third instar larvae in PBS and fixed in 4% formaldehyde/PBS for 15 min. Following fixation, tissues were washed three times in PBT for 5 min each (1% Triton-X100/PBS) and blocked in PBTH (PBT + 5% horse serum) for 1 h. This was followed by incubation in primary antibody, rat anti-HA 1:100 (Sigma-Aldrich, cat# 11867423001) overnight at 4 °C. Subsequent to primary antibody incubation, tissues were washed three times in PBT and incubated in secondary antibody, AlexaFluor594 anti-rat 1:500 (ThermoFisher, cat# A-21209) for 2 h. Tissues were again washed three times in PBT prior to mounting in ProLong Gold Antifade Mountant with DAPI (ThermoFisher, cat# P36935). Unless otherwise stated, all steps were carried out at room temperature. Samples were then imaged on a Zeiss LSM800 Airyscan confocal microscope.

### Co-immunoprecipitation assay

#### Cells

HEK293T cells (CRL-3216) were obtained from ATCC and were maintained in DMEM supplemented with 10% FBS and cultured at 37 °C and 5% CO_2_.

#### Plasmids

Impα1, Impα3, and Impα7 were cloned by PCR into Flag-tagged pCAGGS^52^. pDNR hSOX2 was purchased from Addgene (plasmid #49389; RRID:Addgene_49389) and cloned by PCR into HA-tagged pCAGGS. The HA-tagged pCAGGS SOX2x3Mut was generated by overlapping PCR.

#### Co-immunoprecipitation assay

Flag-tagged Impα1, Impα3, and Impα7 (3 µg), and HA-tagged SOX2 and SOX2x3Mut (3 µg) were transfected into HEK293T cells (1 × 10^6^), using Lipofectamine 2000 (Thermo Fisher Scientific). At 24 h post transfection, cells were lysed in NP-40 lysis buffer (50 mM Tris pH 7.5, 280 mM NaCl, 0.5% NP-40, 0.2 mM EDTA, 2 mM EGTA, 10% glycerol, and protease inhibitor (complete; Roche, Indianapolis, IN)). EZview Red anti-HA agarose affinity gel beads (Sigma-Aldrich) were preincubated in 3% BSA in NP-40 lysis buffer for 1 h at 4 °C. Beads were then incubated with lysates for 30 min at 4 °C, washed ten times in NP-40 lysis buffer, and eluted using HA peptide (Sigma-Aldrich) at 4 °C for 30 min. Whole cell lysates and co-precipitation samples were run on 10% Bis-Tris Plus polyacrylamide gels (ThermoFisher) and transferred to PVDF membrane (Bio-Rad). Membranes were probed with rabbit anti-HA (Invitrogen cat# 71-5500, 1:3000), rabbit anti-Flag (Sigma-Aldrich cat# F7425, 1:3000), mouse anti-β-tubulin (Sigma-Aldrich cat# T8328, 1:10,000), and anti-rabbit IgG HRP-linked antibody (Cell Signaling cat# 7074 S, 1:10,000) or anti-mouse IgG HRP-conjugate antibody (EMD Millipore cat# AP308P, 1:10,000), and were developed by Western Lightening Plus ECL (Perkin Elmer) and imaged on a ChemiDoc MP Imaging System (Bio-Rad).

### Reporting summary

Further information on research design is available in the Nature Research Reporting Summary linked to this article.

## Supplementary information

Supplementary Information

Peer Review File

Reporting Summary

Description of Additional Supplementary Files

Supplementary Movie 1

## Data Availability

The data that support this work are available from the corresponding author upon reasonable request. Protein Data Bank files associated with the structures generated in this study have been deposited to the Protein Data Bank, and issued PDB accession codes 6WX7, 6WX8, and 6WX9.
