# Supplementary material for: Structural basis for nuclear import selectivity of pioneer transcription factor SOX2
Source: Nat Commun. 2021 Jan 4;12:28. doi: 10.1038/s41467-020-20194-0 (PMC7782513; doi:10.1038/s41467-020-20194-0)
Supplement: Supplementary file 1 — Supplementary Information [file 41467_2020_20194_MOESM1_ESM.pdf]

**Supplementary information for STRUCTURAL BASIS FOR NUCLEAR IMPORT SELECTIVITY OF PIONEER TRANSCRIPTION FACTOR SOX2**

Jagga et al

Supplementary Figure 1. The SOX2x3Mut protein is severely impaired in its ability to maintain long-term self-renewal of SOX2-deleted NSC. (A) Cumulative growth curve of SOX2-deleted ($Sox2^{-/-}$) NSC (mutant 2; duplicate of mutant 1 in Figure 4) transduced with wild type SOX2, or SOX2x3Mut, or empty vector (EV). (B) Number of cells recovered at each passage, after plating 20000 cells.

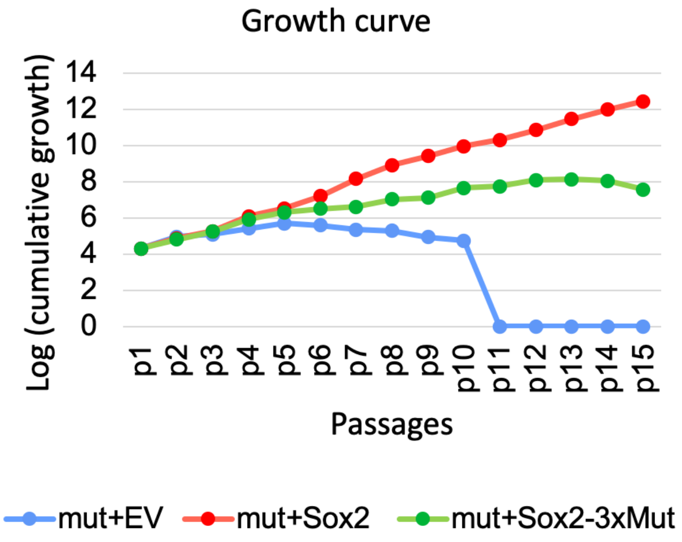


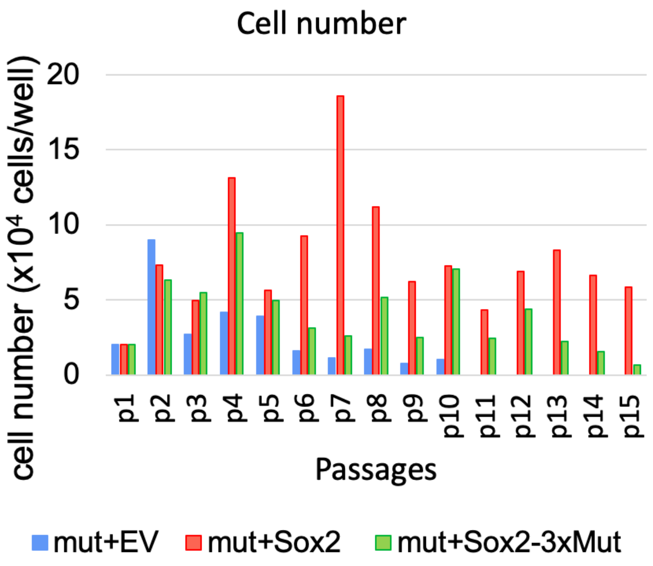

Supplementary Figure 2. FACS analysis of Sox2-deleted cells for GFP expression, marking cells transduced with the indicated vectors, at different passages. A: non transduced mutant (control); B: mutant transduced with empty vector (EV) at passage 1 (p1), passage 2 (p2), passage 3 (p3) and passage 5 (p5); C: mutant transduced with the wild type Sox2 vector, at passages p1, p2, p3, p4, p5 and p13; D: mutant transduced with the Sox2-3x-mut vector, at passages p1, p2, p3, p4, p5 and p13. mut indicates mutant cells (mutant 1). The GFP-positive cells are shown in red. Gating strategy to determine GFP-positive cells is represented in blue (P1 gate) in SSC-A vs. FSC-A dot plots.

A

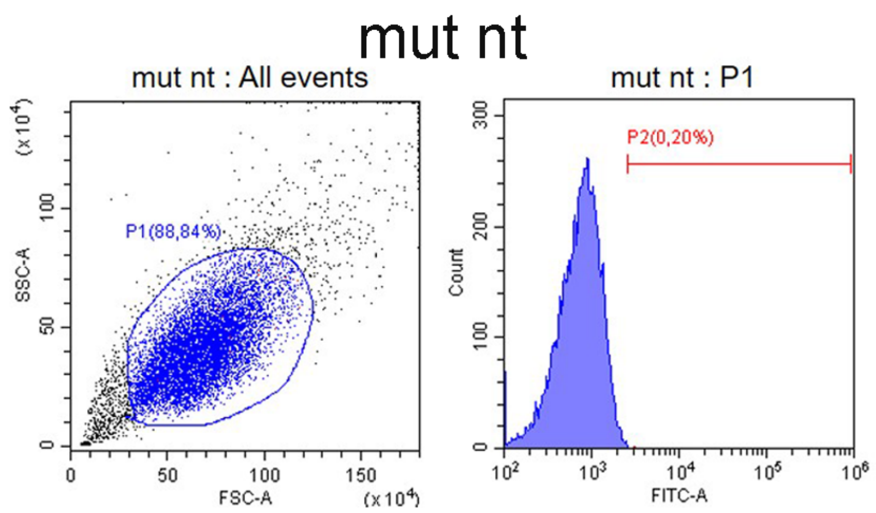

B

mut+EV

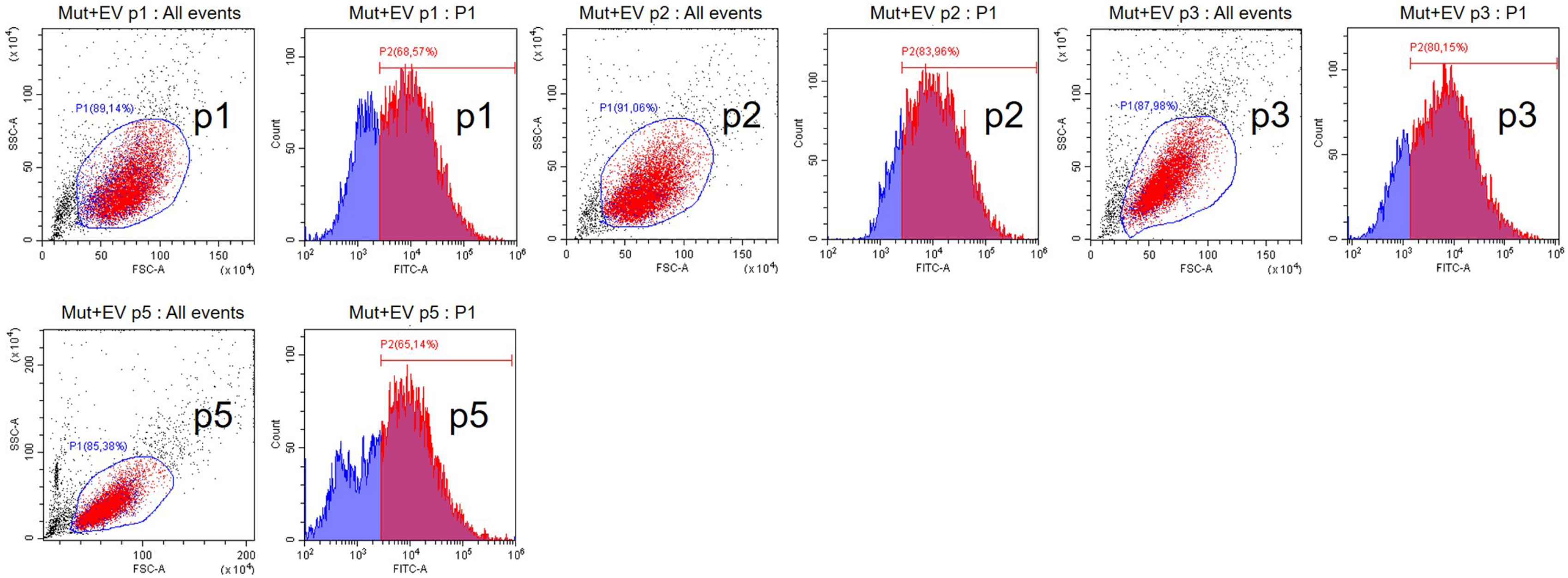

Mut+EV p1 : All events
Mut+EV p1 : P1
Mut+EV p2 : All events
Mut+EV p2 : P1
Mut+EV p3 : All events
Mut+EV p3 : P1
Mut+EV p5 : All events
Mut+EV p5 : P1
P1(89,14%)
P2(68,57%)
P1(91,06%)
P2(83,96%)
P1(87,98%)
P2(80,15%)
P1(85,38%)
P2(65,14%)
p1
p2
p3
p5
SSC-A
FSC-A
Count
FITC-A

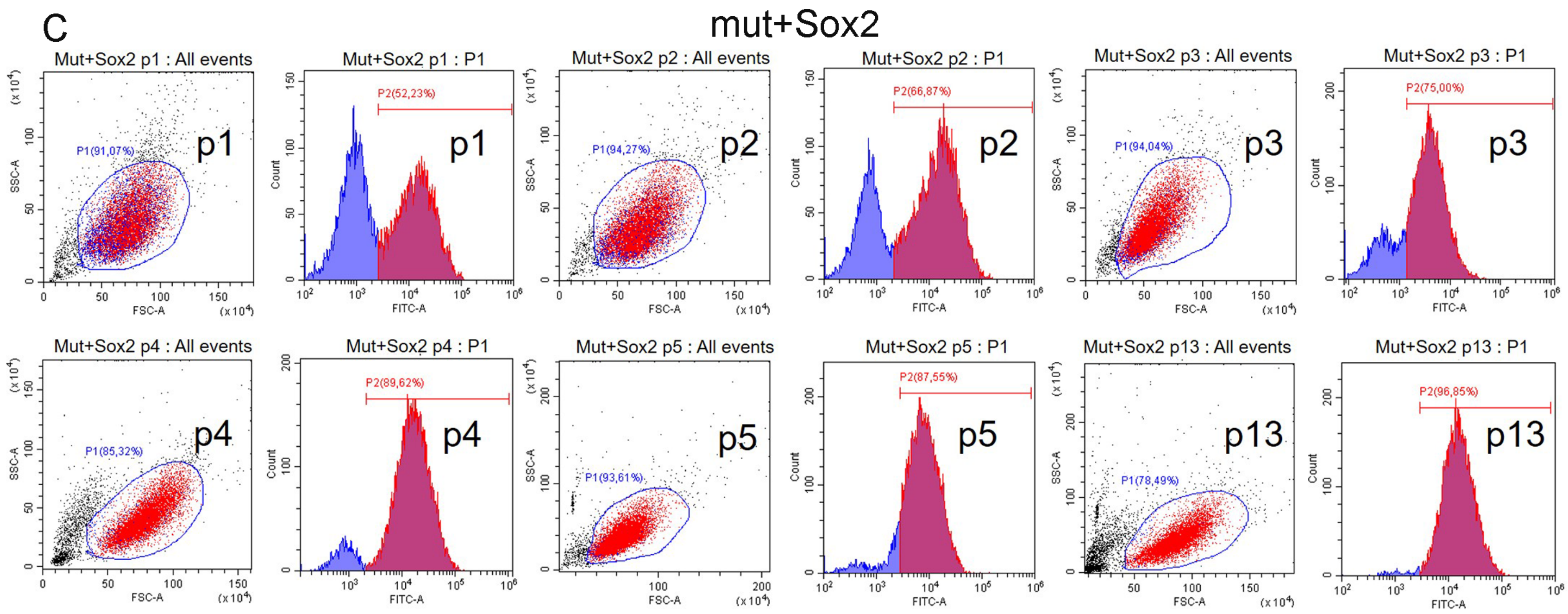
C
mut+Sox2
Mut+Sox2 p1 : All events
Mut+Sox2 p1 : P1
Mut+Sox2 p2 : All events
Mut+Sox2 p2 : P1
Mut+Sox2 p3 : All events
Mut+Sox2 p3 : P1
Mut+Sox2 p4 : All events
Mut+Sox2 p4 : P1
Mut+Sox2 p5 : All events
Mut+Sox2 p5 : P1
Mut+Sox2 p13 : All events
Mut+Sox2 p13 : P1
P1(91,07%)
P2(52,23%)
P1(94,27%)
P2(66,87%)
P1(94,04%)
P2(75,00%)
P1(85,32%)
P2(89,62%)
P1(93,61%)
P2(87,55%)
P1(78,49%)
P2(96,85%)
p1
p2
p3
p4
p5
p13
SSC-A
FSC-A
Count
FITC-A

24

D

mut+Sox2-3x-Mut

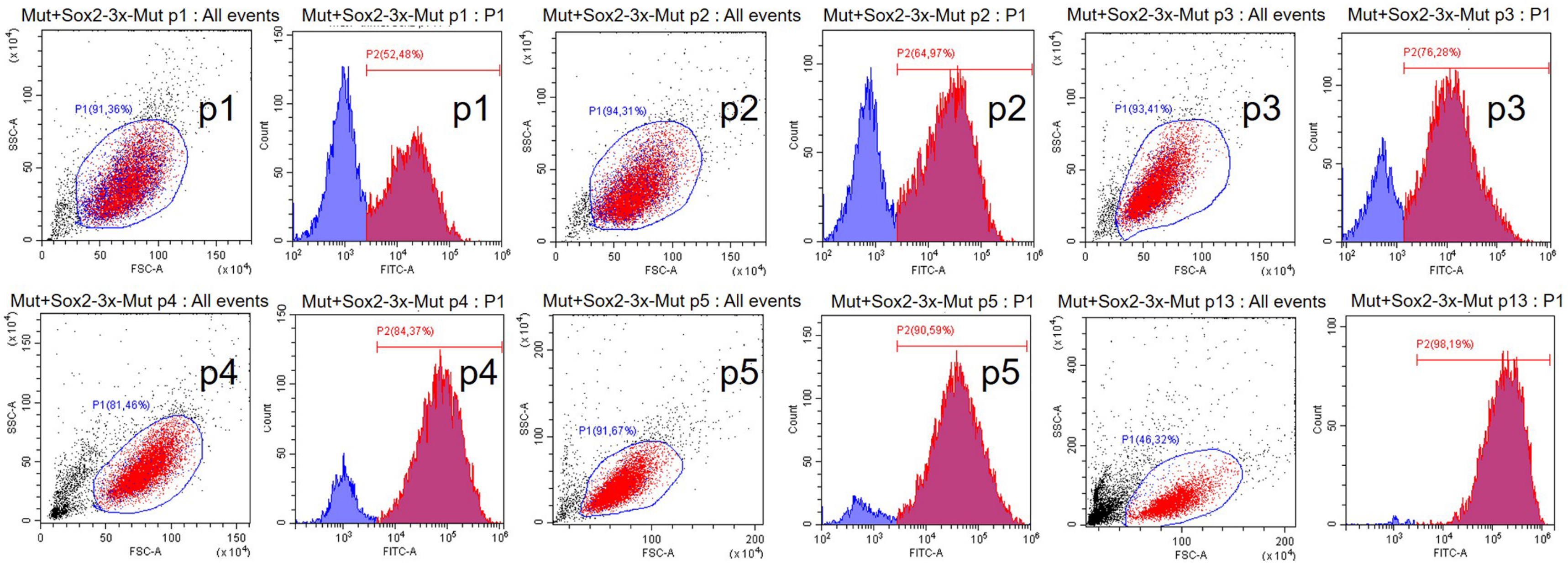

Supplementary Figure 3. Ectopic expression of wildtype and Dichaete3xMut. PtcGal4, UAS-GFP / TM6B was crossed to either UAS-Dichaete.WT / TM6B or UAS-Dichaete.xMut. Ptc-Gal4, UAS-GFP / UAS-Dichaete (WT or 3xMut) progeny numbers were compared with sibling Ptc-Gal4, UAS-GFP or UAS-Dichaete (WT or 3xMut) / TM6B (n = 3, where n represents three independent experiments). Data are presented as mean values +/- SEM. Expression of UAS-Dichaete.WT driven by Ptc-Gal4 results in complete lethality whereas expression of UAS-Dichaete.3xMUT results in survival of adults.

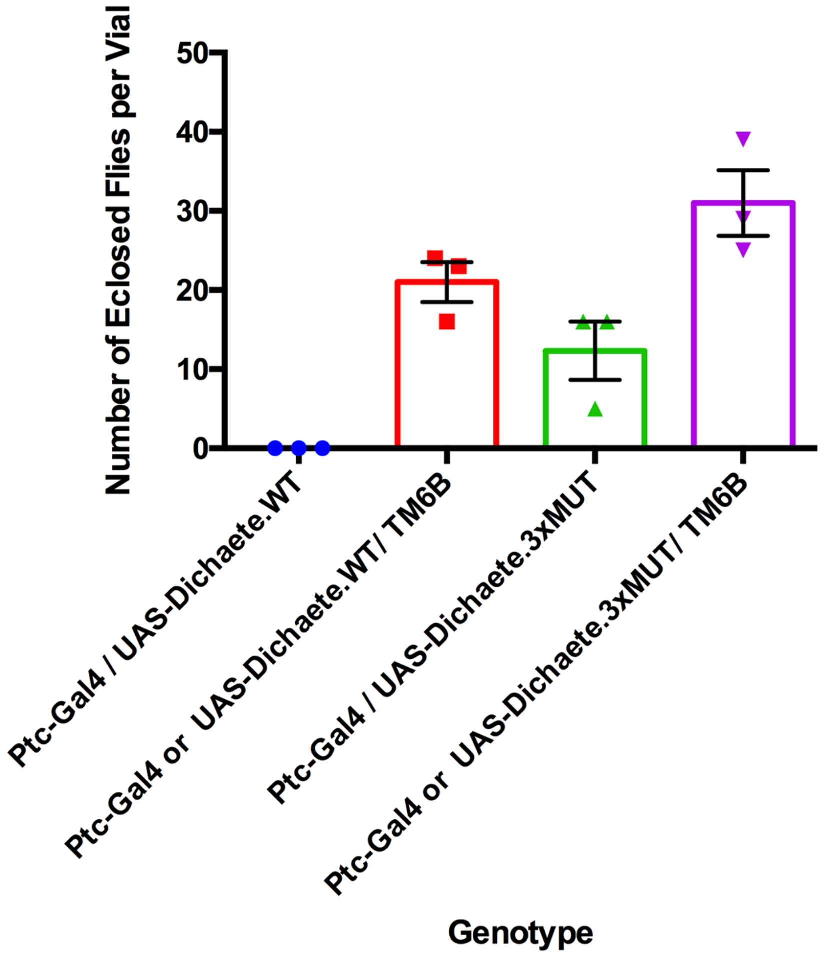

Supplementary Figure 4. Alignment of IMPα isoforms. Residues involved in binding at the IMPα3:SOX2 interface are boxed, and reveal a highly conserved cargo-binding interface.

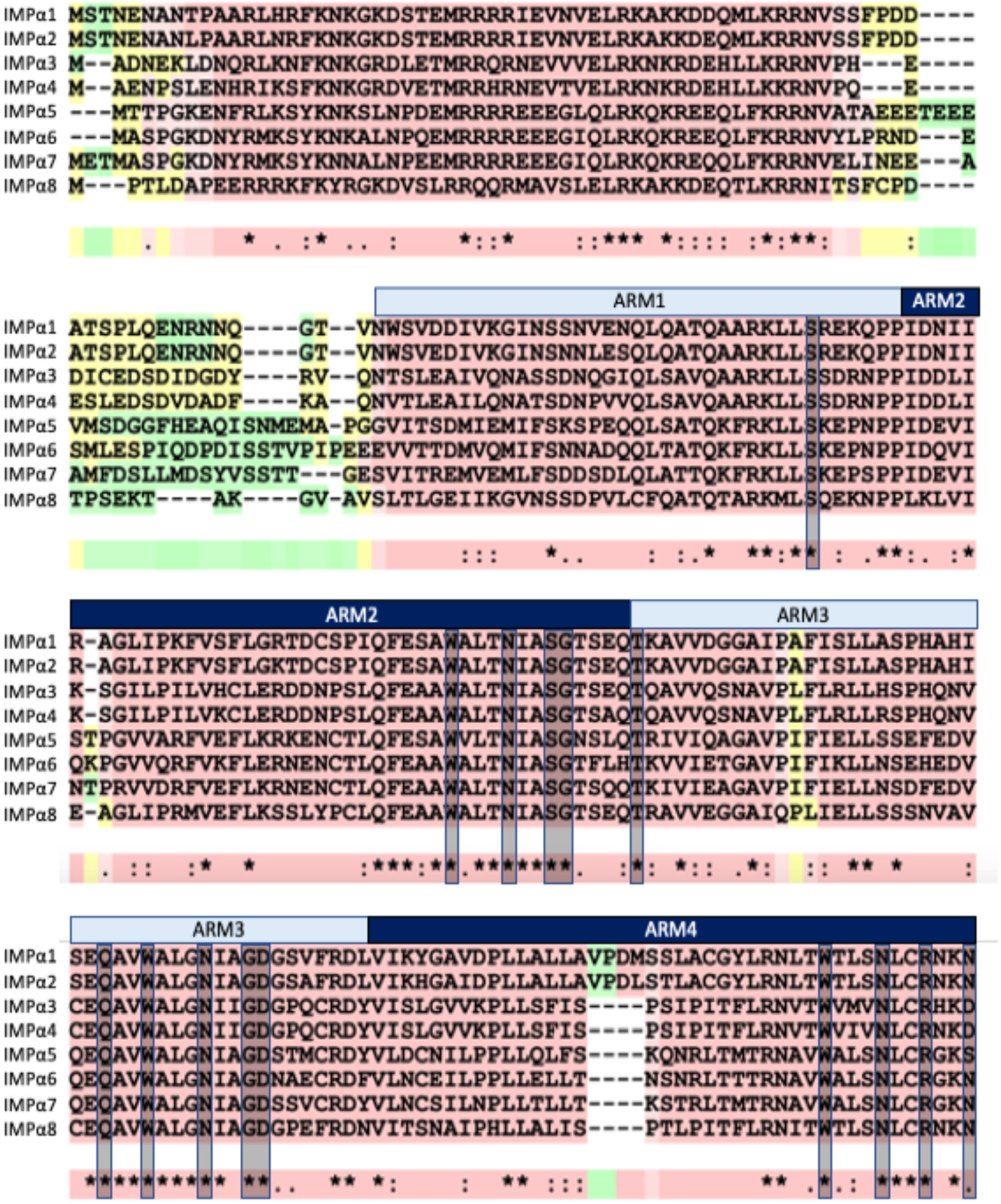

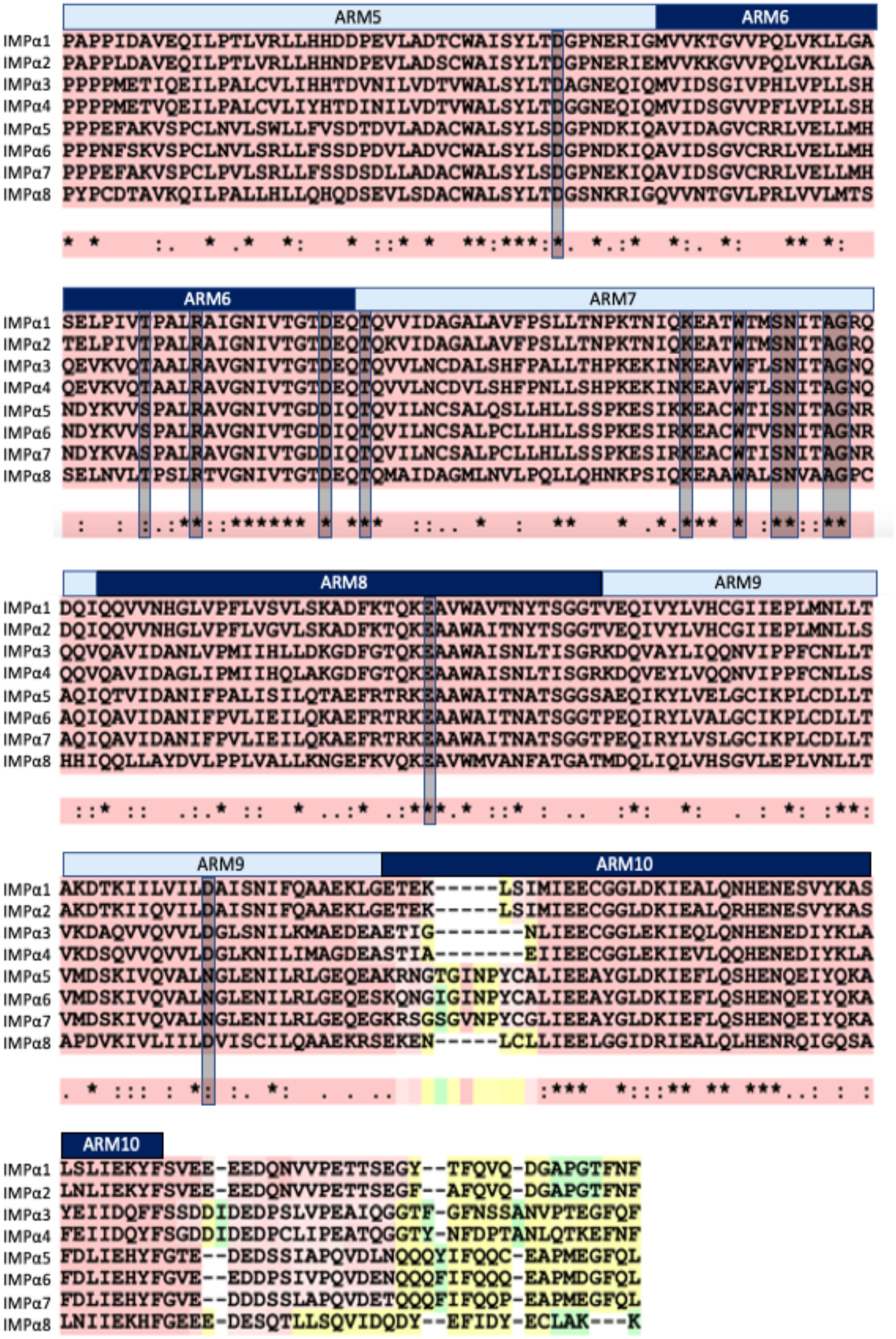
ARM5
ARM6
IMPα1 PAPPIDAVEQILPTLVRLLHHDDPEVLADTCWAISYLTDGPNERIGMVVKTGVVPQLVKLLGA
IMPα2 PAPPLDAVEQILPTLVRLLHHNDPEVLADSCWAISYLTDGPNERIEMVVKKGVVPQLVKLLGA
IMPα3 PPPPMETIQEILPALCVLIHHTDVNILVDTVWALSYLTDAGNEQIQMVIDSGIVPHLVPLLSH
IMPα4 PPPPMETVQEILPALCVLIYHTDINILVDTVWALSYLTDGGNEQIQMVIDSGVVPFLVPLLSH
IMPα5 PPPEFAKVSPCLNVLSWLLFVSDTDVLADACWALSYLSDGPNDKIQAVIDAGVCRRLVELLMH
IMPα6 PPPNFSKVSPCLNVLSRLLFSSDPDVLADVCWALSYLSDGPNDKIQAVIDSGVCRRLVELLMH
IMPα7 PPPEFAKVSPCLPVLSRLLFSSDSDLLADACWALSYLSDGPNEKIQAVIDSGVCRRLVELLMH
IMPα8 PYPCDTAVKQILPALLHLLQHQDSEVLSDACWALSYLTDGSNKRIGQVVNTGVLPRLVVLMTS
ARM6
ARM7
IMPα1 SELPIVTPALRAIGNIVTGTDEQTQVVIDAGALAVFPSLLTNPKTNIQKEATWTMSNITAGRQ
IMPα2 TELPIVTPALRAIGNIVTGTDEQTQKVIDAGALAVFPSLLTNPKTNIQKEATWTMSNITAGRQ
IMPα3 QEVKVQTAALRAVGNIVTGTDEQTQVVLNCDALSHFPALLTHPKEKINKEAVWFLSNITAGNQ
IMPα4 QEVKVQTAALRAVGNIVTGTDEQTQVVLNCDVLSHFPNLLSHPKEKINKEAVWFLSNITAGNQ
IMPα5 NDYKVVSPALRAVGNIVTGDDIQTQVILNCSALQSLLHLLSSPKESIKKEACWTISNITAGNR
IMPα6 NDYKVVSPALRAVGNIVTGDDIQTQVILNCSALPCLLHLLSSPKESIRKEACWTVSNITAGNR
IMPα7 NDYKVASPALRAVGNIVTGDDIQTQVILNCSALPCLLHLLSSPKESIRKEACWTISNITAGNR
IMPα8 SELNVLTPSLRTVGNIVTGTDEQTQMAIDAGMLNVLPQLLQHNKPSIQKEAAWALSNVAAGPC
ARM8
ARM9
IMPα1 DQIQQVVNHGLVPFLVSVLSKADFKTQKEAVWAVTNYTSGGTVEQIVYLVHCGIIEPLMNLLT
IMPα2 DQIQQVVNHGLVPFLVGVLSKADFKTQKEAAWAITNYTSGGTVEQIVYLVHCGIIEPLMNLLS
IMPα3 QQVQAVIDANLVPMIIHLLDKGDFGTQKEAAWAISNLTISGRKDQVAYLIQQNVIPPFCNLLT
IMPα4 QQVQAVIDAGLIPMIIHQLAKGDFGTQKEAAWAISNLTISGRKDQVEYLVQQNVIPPFCNLLS
IMPα5 AQIQTVIDANIFPALISILQTAEFRTRKEAAWAITNATSGGSAEQIKYLVELGCIKPLCDLLT
IMPα6 AQIQAVIDANIFPVLIEILQKAEFRTRKEAAWAITNATSGGTPEQIRYLVALGCIKPLCDLLT
IMPα7 AQIQAVIDANIFPVLIEILQKAEFRTRKEAAWAITNATSGGTPEQIRYLVSLGCIKPLCDLLT
IMPα8 HHIQQLLAYDVLPPLVALLKNGEFKVQKEAVWMVANFATGATMDQLIQLVHSGVLEPLVNLLT
ARM9
ARM10
IMPα1 AKDTKIILVILDAISNIFQAAEKLGETEK-----LSIMIEECGGLDKIEALQNHENESVYKAS
IMPα2 AKDTKIIQVILDAISNIFQAAEKLGETEK-----LSIMIEECGGLDKIEALQRHENESVYKAS
IMPα3 VKDAQVVQVVLDGLSNILKMAEDEAETIG-------NLIEECGGLEKIEQLQNHENEDIYKLA
IMPα4 VKDSQVVQVVLDGLKNILIMAGDEASTIA-------EIIEECGGLEKIEVLQQHENEDIYKLA
IMPα5 VMDSKIVQVALNGLENILRLGEQEAKRNGTGINPYCALIEEAYGLDKIEFLQSHENQEIYQKA
IMPα6 VMDSKIVQVALNGLENILRLGEQESKQNGIGINPYCALIEEAYGLDKIEFLQSHENQEIYQKA
IMPα7 VMDSKIVQVALNGLENILRLGEQEGKRSGSGVNPYCGLIEEAYGLDKIEFLQSHENQEIYQKA
IMPα8 APDVKIVLIILDVISCILQAAEKRSEKEN-----LCLLIEELGGIDRIEALQLHENRQIGQSA
ARM10
IMPα1 LSLIEKYFSVEE-EEDQNVVPETTSEGY--TFQVQ-DGAPGTFNF
IMPα2 LNLIEKYFSVEE-EEDQNVVPETTSEGF--AFQVQ-DGAPGTFNF
IMPα3 YEIIDQFFSSDDIDEDPSLVPEAIQGGTF-GFNSSANVPTEGFQF
IMPα4 FEIIDQYFSGDDIDEDPCLIPEATQGGTY-NFDPTANLQTKEFNF
IMPα5 FDLIEHYFGTE--DEDSSIAPQVDLNQQQYIFQQC-EAPMEGFQL
IMPα6 FDLIEHYFGVE--EDDPSIVPQVDENQQQFIFQQQ-EAPMDGFQL
IMPα7 FDLIEHYFGVE--DDDSSLAPQVDETQQQFIFQQP-EAPMEGFQL
IMPα8 LNIIEKHFGEEE-DESQTLLSQVIDQDY--EFIDY-ECLAK---K

Supplementary Figure 5. The different positioning of ARM domain 7 is constant within each respective IMPα subfamily. The incompatible positioning of SOX2 in ARM 7 of IMPα2 and IMPα5 prevents the full interaction interface observed in IMPα3. Conversely, IMPα3 has not repositioned ARM7 to accommodate the additional interaction interface of SOX2. The reference structures within this study are coloured blue. Structures superimposed on IMPα2 include: PDB 5V5P in cyan, 3UL1 in yellow, 3UKX in green, 4O1H in salmon, and 5B56 in orange. Structures superimposed on IMPα3 include: PDB 5TBK in cyan, 6BW9 yellow, 4UAI in green, 5XZX in salmon, and 6BVV in orange. IMPα5 include: 4B18 in cyan, 3TJ3 in yellow, and 2JDQ in green.

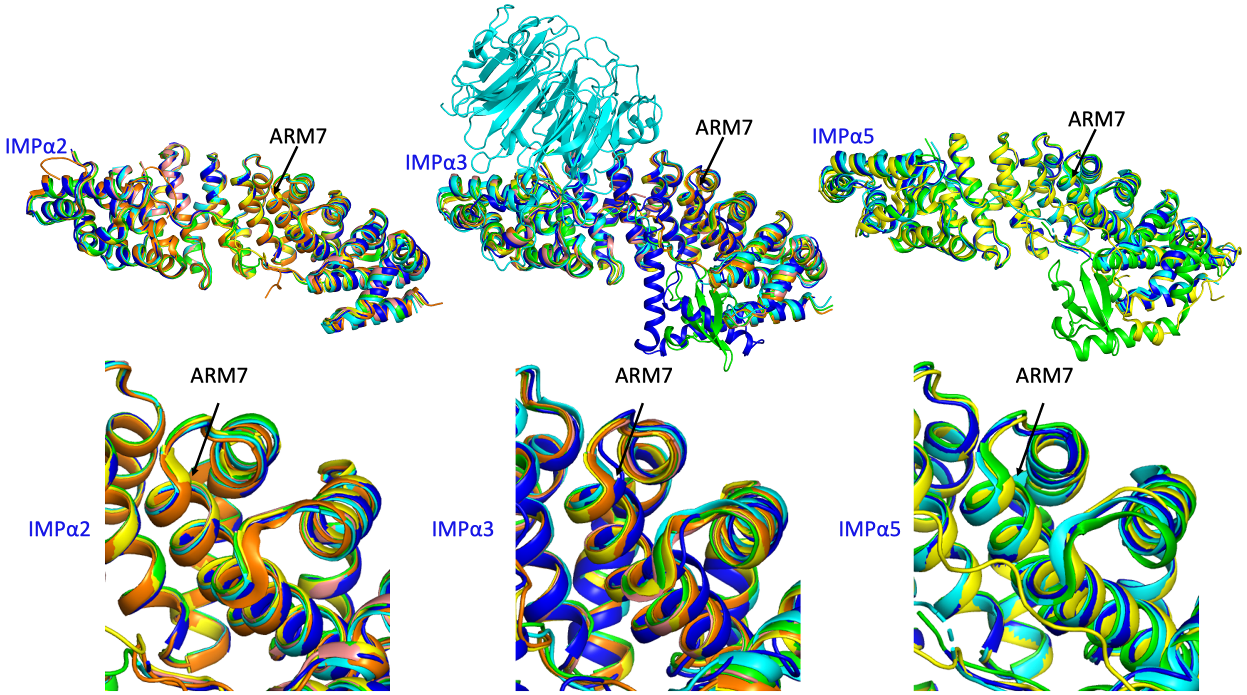

Supplementary Figure 6. Uncropped gels for figures presented in Figure 3A.

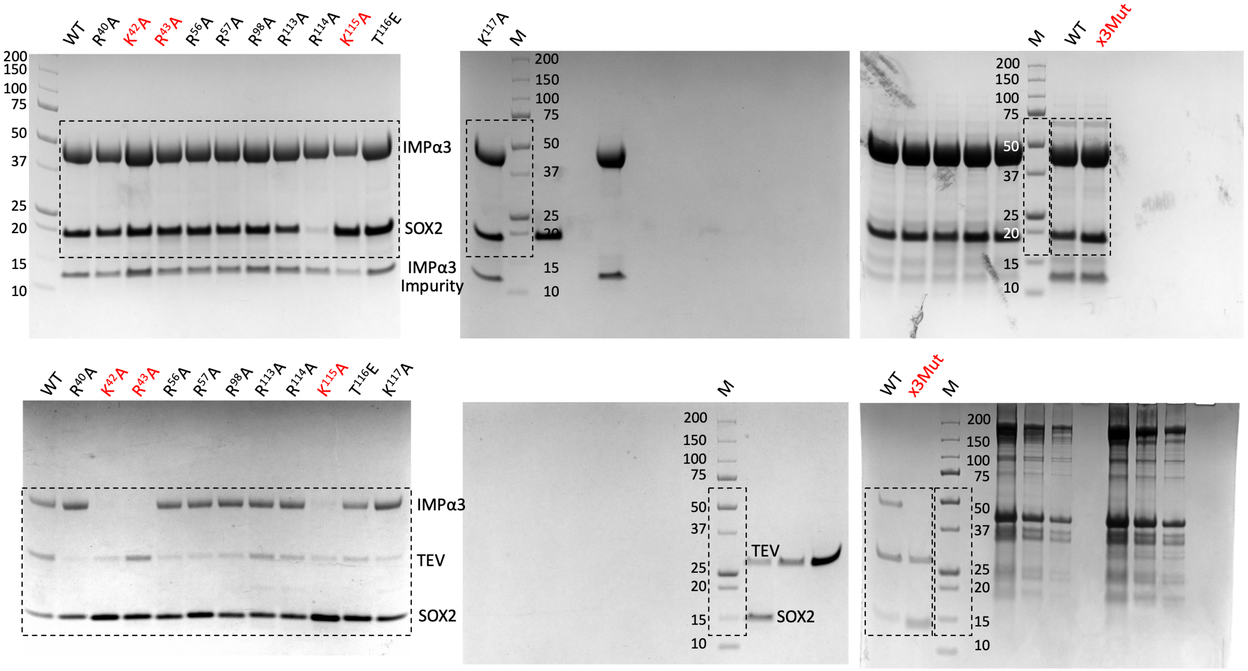

Supplementary Figure 7. Distribution of data points for Figure 4f.

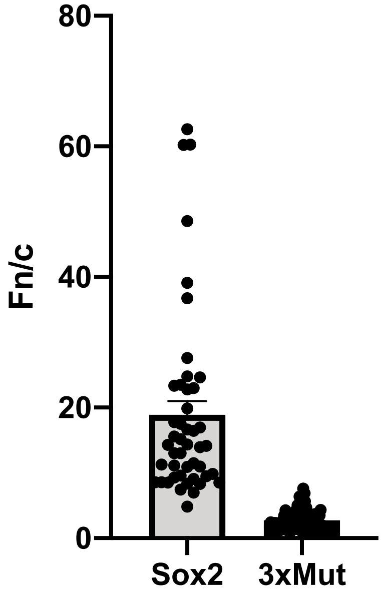

Supplementary Figure 8. Distribution of data points shown in Figure 5. Quantification of fluorescence in the nucleus compared to the cytoplasm (expressed as Fn/c). Salivary gland cells (left), salivary duct cells (right).

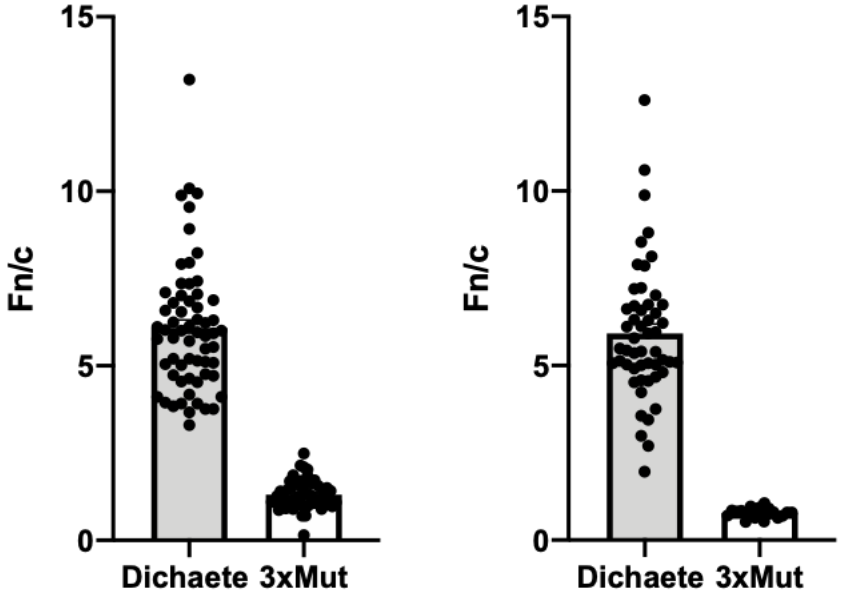

Supplementary Figure 9. Uncropped gels for Western blot presented in Figure 6A. Western blots for the whole cell lysates were run in duplicate in parallel; one membrane was first probed with anti-Flag to detect Flag-tagged IMPA followed by anti-HA to detect HA-tagged Sox2. The second membrane was probed for b-tubulin.

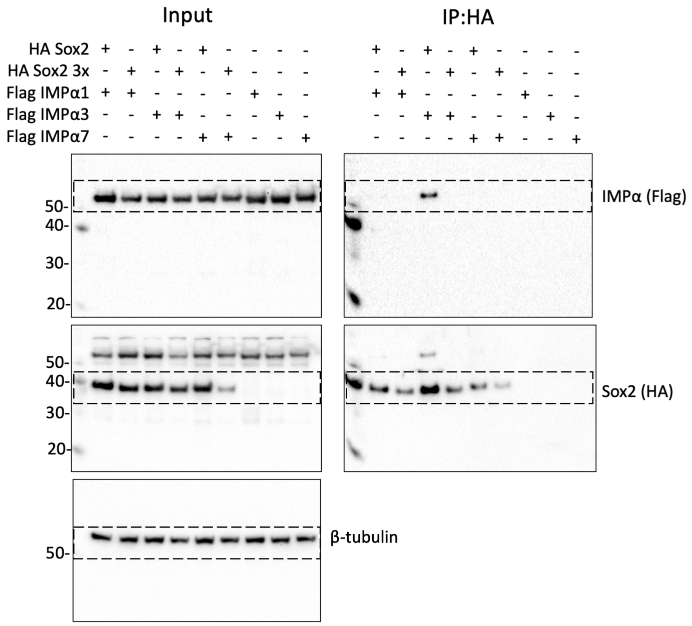

Supplementary Figure 10. Uncropped gels for figures presented in Figure 6B.

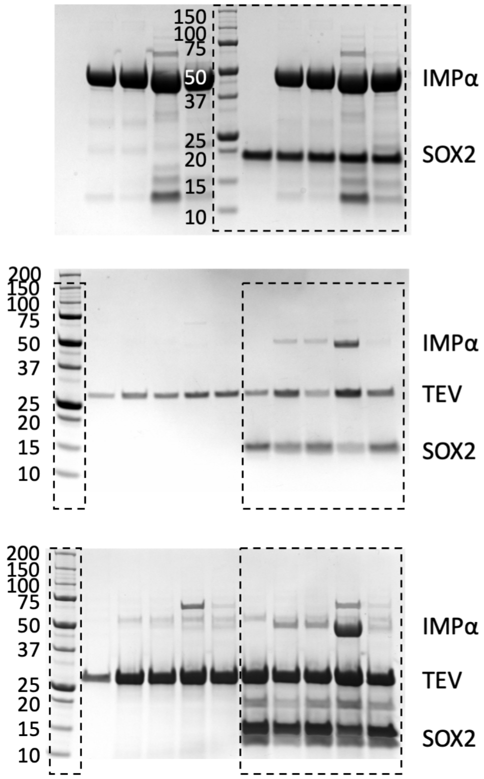

Supplementary Table 1. Data collection and refinement statistics.

| Data collection and processing | SOX2:IMPα2 | SOX2:IMPα3 | SOX2:IMPα5 |
|---|---|---|---|
| Wavelength (Å) | 0.9537 | 0.9537 | 0.9537 |
| Resolution range (Å) | 29.1-2.7 (2.8-2.7) | 29.5-2.3 (2.4-2.3) | 29.1-2.8 (2.9-2.8) |
| Space group | P 21 21 21 | C 1 2 1 | C 1 2 1 |
| Unit cell (Å, $^{o}$) | 78.1 91.3 97.6 90 90 90 | 148.6 119.0 94.9 90 128.8 90 | 160.5 61.4 69.1 90 95.5 90 |
| Total reflections | 193418 (19377) | 740984 (50390) | 51070 (7643) |
| Unique reflections | 18955 (2235) | 56880 (4570) | 16476 (2377) |
| Multiplicity | 10.2 (8.2) | 13.0 (11.0) | 3.1 (3.2) |
| Completeness (%) | 96.4 (87.90) | 99.6 (97.8) | 98.8 (99.0) |
| Mean I/sigma(I) | 11.2 (2.2) | 12.6 (2.0) | 6.5 (1.8) |
| Wilson B-factor Å$^2$ | 29.1 | 51.8 | 60.9 |
| R-merge % | 15.2 (96.1) | 10.7 (136.9) | 8.0 (55.4) |
| R-pim % | 4.9 (33.3) | 3.2 (44.8) | 5.8 (42.7) |
| Refinement | | | |
| Number of reflections | 18926 | 52545 | 12841 |
| Number of R-free reflections | 896 | 2747 | 626 |
| R-work % | 19.5 | 22.0 | 20.5 |
| R-free % | 22.7 | 24.5 | 25.2 |
| RMS(bonds) | 0.002 | 0.002 | 0.004 |
| RMS(angles) | 0.53 | 0.49 | 0.66 |
| Ramachandran plot | | | |
| favored (%) | 97.9 | 99.0 | 97.7 |
| allowed (%) | 2.1 | 1.0 | 2.3 |
| outliers (%) | 0 | 0 | 0 |
| Validation | | | |
| Clash score | 1.5 | 3.0 | 10.6 |
| MolProbity score (percentile) | 0.9 (100) | 1.2 (100) | 1.54 (100) |
| PDB accession code | 6WX7 | 6WX8 | 6WX9 |

Statistics for the highest-resolution shell are shown in parentheses.

Supplementary Table 2. Summary of IMPα2:SOX2 interactions

| Hydrogen Bonds | | | |
|---|---|---|---|
| # | SOX2 | Dist. (Å) | IMPα2 |
| 1 | B:LYS 42[ NZ ] | 3.20 | A:GLY 323[ O ] |
| 2 | B:LYS 42[ NZ ] | 2.69 | A:VAL 321[ O ] |
| 3 | B:LYS 42[ NZ ] | 3.20 | A:THR 328[ OG1] |
| 4 | B:ARG 43[ N ] | 2.97 | A:ASN 361[ OD1] |
| 5 | B:ARG 43[ NH1] | 3.17 | A:GLU 396[ OE1] |
| 6 | B:ARG 43[ NH1] | 3.81 | A:SER 360[ OG ] |
| 7 | B:ARG 43[ NH2] | 3.74 | A:GLU 396[ OE2] |
| 8 | B:ARG 113[ NH2] | 3.62 | A:ASN 239[ OD1] |
| 9 | B:ARG 114[ NH1] | 2.21 | A:GLY 150[ O ] |
| 10 | B:ARG 114[ NH2] | 3.52 | A:SER 152[ OG ] |
| 11 | B:ARG 114[ NH2] | 2.34 | A:ASP 192[ OD1] |
| 12 | B:LYS 115[ N ] | 2.90 | A:ASN 188[ OD1] |
| 13 | B:LYS 117[ N ] | 3.36 | A:ASN 146[ OD1] |
| 14 | B:LYS 117[ NZ ] | 3.12 | A:GLN 181[ OE1] |
| 15 | B:ARG 43[ O ] | 3.04 | A:TRP 357[ NE1] |
| 16 | B:ARG 43[ O ] | 3.40 | A:ASN 361[ ND2] |
| 17 | B:ARG 111[ O ] | 3.15 | A:ARG 238[ NH1] |
| 18 | B:ARG 111[ O ] | 3.41 | A:ARG 238[ NH2] |
| 19 | B:PRO 112[ O ] | 3.20 | A:TRP 231[ NE1] |
| 20 | B:ARG 113[ O ] | 3.10 | A:ASN 235[ ND2] |
| 21 | B:LYS 115[ O ] | 2.85 | A:ASN 188[ ND2] |
| 22 | B:LYS 115[ O ] | 3.02 | A:TRP 184[ NE1] |
| 23 | B:LYS 117[ O ] | 3.01 | A:ASN 146[ ND2] |
| 24 | B:LYS 117[ O ] | 3.31 | A:TRP 142[ NE1] |
| Salt Bridges | | | |
| # | SOX2 | Dist. (Å) | IMPα2 |
| 1 | B:ARG 43[ NH1] | 4.00 | A:GLU 396[ OE2] |
| 2 | B:ARG 43[ NH1] | 3.17 | A:GLU 396[ OE1] |
| 3 | B:ARG 43[ NH2] | 3.74 | A:GLU 396[ OE2] |
| 4 | B:ARG 114[ NH2] | 2.34 | A:ASP 192[ OD1] |

Supplementary Table 3. Summary of IMPα3:SOX2 interactions

| Hydrogen Bonds | | | |
|---|---|---|---|
| # | SOX2 | Dist. (Å) | IMPα3 |
| 1 | D:ARG 40[ NE ] | 2.85 | C:ASP 316[ OD2] |
| 2 | D:ARG 40[ NH1] | 2.99 | C:GLY 356[ O ] |
| 3 | D:ARG 40[ NH1] | 3.70 | C:ALA 355[ O ] |
| 4 | D:LYS 42[ NZ ] | 3.55 | C:THR 319[ OG1] |
| 5 | D:LYS 42[ NZ ] | 2.74 | C:ASN 352[ O ] |
| 6 | D:ARG 43[ N ] | 2.91 | C:ASN 352[ OD1] |
| 7 | D:ARG 43[ NH1] | 3.06 | C:SER 351[ OG ] |
| 8 | D:ARG 43[ NH2] | 2.26 | C:GLU 387[ OE2] |
| 9 | D:ARG 57[ NH2] | 2.38 | C:ASP 433[ OD2] |
| 10 | D:LYS 95[ NZ ] | 3.57 | C:ASP 232[ OD2] |
| 11 | D:ARG 98[ NH2] | 2.91 | C:ASP 271[ OD2] |
| 12 | D:ARG 114[ NH1] | 2.97 | C:GLY 186[ O ] |
| 13 | D:ARG 114[ NH1] | 3.77 | C:ASP 187[ O ] |
| 14 | D:LYS 115[ NZ ] | 3.09 | C:GLY 145[ O ] |
| 15 | D:LYS 115[ NZ ] | 2.89 | C:THR 150[ OG1] |
| 16 | D:LYS 115[ NZ ] | 3.02 | C:ASP 187[ OD1] |
| 17 | D:THR 116[ N ] | 2.90 | C:ASN 183[ OD1] |
| 18 | D:LYS 117[ NZ ] | 3.47 | C:SER 144[ OG ] |
| 19 | D:THR 118[ N ] | 2.90 | C:ASN 141[ OD1] |
| 20 | D:MET 120[ N ] | 3.26 | C:SER 100[ O ] |
| 21 | D:ARG 43[ O ] | 2.99 | C:ASN 352[ ND2] |
| 22 | D:ARG 43[ O ] | 2.81 | C:TRP 348[ NE1] |
| 23 | D:HIS 101[ O ] | 2.80 | C:ARG 306[ NH2] |
| 24 | D:GLU 104[ OE1] | 3.72 | C:LYS 344[ NZ ] |
| 25 | D:ASP 107[ OD2] | 2.51 | C:THR 302[ OG1] |
| 26 | D:PRO 112[ O ] | 2.33 | C:ARG 229[ NH2] |
| 27 | D:ARG 113[ O ] | 2.79 | C:TRP 222[ NE1] |
| 28 | D:ARG 114[ O ] | 2.84 | C:ASN 226[ ND2] |
| 29 | D:THR 116[ O ] | 3.31 | C:TRP 179[ NE1] |
| 30 | D:THR 116[ O ] | 2.94 | C:ASN 183[ ND2] |
| 31 | D:THR 116[ OG1] | 2.72 | C:TRP 179[ NE1] |
| 32 | D:THR 118[ O ] | 2.71 | C:TRP 137[ NE1] |
| 33 | D:THR 118[ O ] | 3.20 | C:ASN 141[ ND2] |
| 34 | D:THR 118[ OG1] | 3.26 | C:GLN 176[ NE2] |
| Salt Bridges | | | |
| # | SOX2 | Dist. (Å) | IMPα3 |
| 1 | D:ARG 40[ NE ] | 3.53 | C:ASP 316[ OD1] |
| 2 | D:ARG 40[ NE ] | 2.85 | C:ASP 316[ OD2] |
| 3 | D:ARG 40[ NH2] | 3.48 | C:ASP 316[ OD2] |
| 4 | D:ARG 43[ NH2] | 3.56 | C:GLU 387[ OE1] |
| 5 | D:ARG 43[ NH2] | 2.26 | C:GLU 387[ OE2] |
| 6 | D:ARG 57[ NH1] | 3.59 | C:ASP 433[ OD1] |
| 7 | D:ARG 57[ NH1] | 3.27 | C:ASP 433[ OD2] |

| | | | |
|---|---|---|---|
| 8 | D:ARG 57[ NH2] | 3.72 | C:ASP 433[ OD1] |
| 9 | D:ARG 57[ NH2] | 2.38 | C:ASP 433[ OD2] |
| 10 | D:LYS 95[ NZ ] | 3.57 | C:ASP 232[ OD2] |
| 11 | D:ARG 98[ NE ] | 3.26 | C:ASP 271[ OD2] |
| 12 | D:ARG 98[ NH2] | 2.91 | C:ASP 271[ OD2] |
| 13 | D:LYS 115[ NZ ] | 3.02 | C:ASP 187[ OD1] |
| 14 | D:GLU 104[ OE1] | 3.72 | C:LYS 344[ NZ ] |

Supplementary Table 4. Summary of IMPα5:SOX2 interactions

| Hydrogen Bonds | | | |
|---|---|---|---|
| # | SOX2 | Dist. (Å) | IMPα5 |
| 1 | B:LYS 42[ NZ ] | 2.78 | A:VAL 324[ O ] |
| 2 | B:LYS 42[ NZ ] | 2.56 | A:GLY 326[ O ] |
| 3 | B:ARG 43[ N ] | 3.15 | A:ASN 364[ OD1] |
| 4 | B:ARG 43[ NH2] | 2.41 | A:GLU 399[ OE2] |
| 5 | B:ARG 43[ NH2] | 3.04 | A:SER 363[ OG ] |
| 6 | B:ARG 114[ NE ] | 3.31 | A:ALA 155[ O ] |
| 7 | B:ARG 114[ NE ] | 3.53 | A:ASN 195[ O ] |
| 8 | B:ARG 114[ NH1] | 2.52 | A:GLY 157[ O ] |
| 9 | B:ARG 114[ NH2] | 3.77 | A:ASN 158[ O ] |
| 10 | B:ARG 114[ NH2] | 2.52 | A:ASP 199[ OD1] |
| 11 | B:LYS 115[ N ] | 2.94 | A:ASN 195[ OD1] |
| 12 | B:THR 116[ OG1] | 3.65 | A:SER 156[ OG ] |
| 13 | B:LYS 117[ N ] | 3.11 | A:ASN 153[ OD1] |
| 14 | B:LYS 117[ NZ ] | 2.52 | A:GLN 188[ OE1] |
| 15 | B:ARG 43[ O ] | 3.12 | A:TRP 360[ NE1] |
| 16 | B:ARG 43[ O ] | 3.32 | A:ASN 364[ ND2] |
| 17 | B:PRO 44[ O ] | 3.59 | A:TRP 360[ NE1] |
| 18 | B:MET 45[ SD ] | 2.63 | A:ARG 318[ NH1] |
| 19 | B:ARG 113[ O ] | 3.22 | A:ASN 238[ ND2] |
| 20 | B:LYS 115[ O ] | 3.23 | A:ASN 195[ ND2] |
| 21 | B:LYS 115[ O ] | 3.06 | A:TRP 191[ NE1] |
| 22 | B:LYS 117[ O ] | 3.25 | A:TRP 149[ NE1] |
| 23 | B:LYS 117[ O ] | 3.24 | A:ASN 153[ ND2] |
| Salt Bridges | | | |
| # | SOX2 | Dist. (Å) | IMPα5 |
| 1 | B:ARG 43[ NH1] | 3.13 | A:GLU 399[ OE2] |
| 2 | B:ARG 43[ NH2] | 2.41 | A:GLU 399[ OE2] |
| 3 | B:ARG 114[ NH1] | 3.61 | A:ASP 199[ OD1] |
| 4 | B:ARG 114[ NH2] | 2.52 | A:ASP 199[ OD1] |
