# Supplementary material for: Structural basis for nuclear import selectivity of pioneer transcription factor SOX2
Source: Nat Commun. 2021 Jan 4;12:28. doi: 10.1038/s41467-020-20194-0 (PMC7782513; doi:10.1038/s41467-020-20194-0)
Supplement: Supplementary file 3 — Reporting Summary [file 41467_2020_20194_MOESM3_ESM.pdf]

nature research

Corresponding author(s): Jade Forwood

Last updated by author(s): Sep 18, 2020

Reporting Summary

Nature Research wishes to improve the reproducibility of the work that we publish. This form provides structure for consistency and transparency in reporting. For further information on Nature Research policies, see our Editorial Policies and the Editorial Policy Checklist.

Statistics

For all statistical analyses, confirm that the following items are present in the figure legend, table legend, main text, or Methods section.

n/a	Confirmed	
☐	☒	The exact sample size (*n*) for each experimental group/condition, given as a discrete number and unit of measurement
☐	☒	A statement on whether measurements were taken from distinct samples or whether the same sample was measured repeatedly
☒	☐	The statistical test(s) used AND whether they are one- or two-sided<br>*Only common tests should be described solely by name; describe more complex techniques in the Methods section.*
☒	☐	A description of all covariates tested
☒	☐	A description of any assumptions or corrections, such as tests of normality and adjustment for multiple comparisons
☒	☐	A full description of the statistical parameters including central tendency (e.g. means) or other basic estimates (e.g. regression coefficient) AND variation (e.g. standard deviation) or associated estimates of uncertainty (e.g. confidence intervals)
☐	☒	For null hypothesis testing, the test statistic (e.g. *F*, *t*, *r*) with confidence intervals, effect sizes, degrees of freedom and *P* value noted<br>*Give P values as exact values whenever suitable.*
☒	☐	For Bayesian analysis, information on the choice of priors and Markov chain Monte Carlo settings
☒	☐	For hierarchical and complex designs, identification of the appropriate level for tests and full reporting of outcomes
☒	☐	Estimates of effect sizes (e.g. Cohen's *d*, Pearson's *r*), indicating how they were calculated

Our web collection on statistics for biologists contains articles on many of the points above.

Software and code

Policy information about availability of computer code

Data collection	iMosflm version 7.30; AIMLESS and Phaser were used from within the CCP4 release version 7.1.005; Coot version 0.9; UCLA MBI server (https://services.mbi.ucla.edu/anisoscale/) version 1.2; Phenix refine within Phenix release version 1.14-3260; Nanotemper Technoloogies analysis software version 1.5.41
Data analysis	iMosflm version 7.30; AIMLESS and Phaser were used from within the CCP4 release version 7.1.005; Coot version 0.9; UCLA MBI server (https://services.mbi.ucla.edu/anisoscale/) version 1.2; Phenix refine within Phenix release version 1.14-3260; Nanotemper Technoloogies analysis software version 1.5.41

For manuscripts utilizing custom algorithms or software that are central to the research but not yet described in published literature, software must be made available to editors and reviewers. We strongly encourage code deposition in a community repository (e.g. GitHub). See the Nature Research guidelines for submitting code & software for further information.

Data

Policy information about availability of data

All manuscripts must include a data availability statement. This statement should provide the following information, where applicable:

- Accession codes, unique identifiers, or web links for publicly available datasets
- A list of figures that have associated raw data
- A description of any restrictions on data availability

PDB Accession codes: 6WX7 6WX8 6WX9

Field-specific reporting

Please select the one below that is the best fit for your research. If you are not sure, read the appropriate sections before making your selection.

☒ Life sciences ☐ Behavioural & social sciences ☐ Ecological, evolutionary & environmental sciences

For a reference copy of the document with all sections, see nature.com/documents/nr-reporting-summary-flat.pdf

Life sciences study design

All studies must disclose on these points even when the disclosure is negative.

Sample size	Figure 3B: An n value of 3 was used. This is standard in the field to use triplicates for quantitative binding measurements. The sample size was sufficient comparing the binding affinity data.<br>Figure 4F. The n-value used is 46 and 96 independent cells for the SOX2 and SOX2x3Mut samples respectively. This was based on the number of cells available, and was a large enough dataset to obtain statistically significant differences between the two samples.<br>Figure 5. The n-value used is 62 and 51 (salivary gland); 52 and 20 (salivary duct) independent cells for the HA-Dichaete and HA-Dichaete-3xMut samples respectively. This was based on the number of cells available, and was a large enough dataset to obtain statistically significant differences between the two samples.<br>Figure 6. An n value of 3 was used. This is standard in the field to use triplicates for quantitative binding measurements. The sample size was sufficient for comparing the binding affinity data.
Data exclusions	No data was excluded
Replication	All attempts at replication were successful and performed in triplicate.
Randomization	The laboratory procedures performed in this study such as structural characterisation of protein complexes using X-ray crystallographic approaches, and standard biochemical analysis do not require randomisation to be implemented in the data collection. Randomisation would be a very costly, cumbersome, and unnecessary way to perform these experiments.
Blinding	The laboratory procedures performed in this study such as structural characterisation of protein complexes using X-ray crystallographic approaches, and standard biochemical analysis do not require blinding to be implemented in the data collection. Blinding would be a very costly, cumbersome, and unnecessary way to perform these experiments.

Reporting for specific materials, systems and methods

We require information from authors about some types of materials, experimental systems and methods used in many studies. Here, indicate whether each material, system or method listed is relevant to your study. If you are not sure if a list item applies to your research, read the appropriate section before selecting a response.

Materials & experimental systems

n/a	Involved in the study	
☐	☒	Antibodies
☐	☒	Eukaryotic cell lines
☒	☐	Palaeontology and archaeology
☐	☒	Animals and other organisms
☒	☐	Human research participants
☒	☐	Clinical data
☒	☐	Dual use research of concern

Methods

n/a	Involved in the study	
☒	☐	ChIP-seq
☐	☒	Flow cytometry
☒	☐	MRI-based neuroimaging

Antibodies

Antibodies used	>Rabbit Anti-FLAG antibody, Sigma Aldrich, Catalog Number F7425<br>>Rabbit HA Tag Polyclonal Antibody, Invitrogen, Catalog Number 71-5500<br>>Mouse Anti-β-Tubulin antibody, Sigma Aldrich, Catalog Number T8328<br>>Anti rabbit IgG HRP linked Antibody, Cell Signaling, Catalog Number 7074S<br>>Anti-Mouse IgG HRP conjugate Antibody, EMD Millipore, Catalog Number AP308P<br>>Rat anti-HA IgG1 monoclonal clone 3F10, Sigma Aldrich, Catalog Number: 11867423001<br>>AlexaFluor594 anti-rat, ThermoFisher, Catalog Number A-21209<br>>Anti-h/m/rSOX2: purified mouse monoclonal IgG2A, R&D System, Clone 245610, Catalog number MAB2018, Lot: KGQ0319101,<br>>Anti-SOX2 antibody, secondary rhodamine (TRITC)-conjugated goat anti-mouse IgG2a antibodies<br>>Goat anti-Mouse IgG2a Cross-Adsorbed Secondary Antibody, Alexa Fluor 594, ThermoFisher Scientific, Catalog Number A-21135.
Validation	>Rabbit ANTI-FLAG antibody, Sigma Aldrich, cat# F7425. Sigma Aldrich states that the rabbit Anti-FLAG polyclonal affinity antibody

ANTI-FLAG recognizes the FLAG epitope located on FLAG fusion proteins and reacts with N-terminal, N-terminal-Met, and C-terminal FLAG fusion proteins.
>Rabbit HA Tag Polyclonal Antibody, Invitrogen, cat# 71-5500. Invitrogen states that the rabbit anti-HA antibody was generated to the HA tag and reactivity was confirmed to the HA tag. In addition, the data in Fig. 6a demonstrate that the anti-HA antibody does not significantly react on western blot with proteins in our lysates when an HA-tagged protein is not expressed.
>Mouse Anti-β-Tubulin antibody, Sigma Aldrich, cat# T8328. Sigma Aldrich states mouse anti-β-tubulin antibody reacts specifically with β tubulin, types I, II, III, and IV of bovine, rat, mouse and human.
>Rat anti-HA IgG1 monoclonal clone 3F10, Sigma Aldrich, Catalog Number: 11867423001. Recognises HA-peptide (YPYDVPDYA) derived from human influenza hemagglutinin protein. Western blot available at supplier website: https://www.sigmaaldrich.com/catalog/product/roche/roahaha?lang=en&region=AU&gclid=CjwKCAjwkoz7BRBPEiwAeKw3qxG_qGKptSQ08591W98gl1ICbvS71LdFFmABfSekuvD3mgvCudC5sBoC5GsQAvD_BwE
>Anti-h/m/rSOX2: purified mouse monoclonal IgG2A, R&D System, Clone 245610, Catalog number MAB2018, Lot: KGQ0319101, https://www.rndsystems.com/products/human-mouse-rat-sox2-antibody-245610_mab2018. Validation blots and references are available from the website.
>Goat anti-Mouse IgG2a Cross-Adsorbed Secondary Antibody, Alexa Fluor 594, ThermoFisher Scientific
Catalog #A-21135. https://www.thermofisher.com/antibody/product/Goat-anti-Mouse-IgG2a-Cross-Adsorbed-Secondary-Antibody-Polyclonal/A-21135. The antibody was validated for not showing any reactivity with Sox2-deleted mouse neural cells, in Cavallaro M et al., Development 2008 (PMID: 18171687)

Eukaryotic cell lines

Policy information about cell lines

Cell line source(s)
HEK293T cells (CRL-3216) were obtained from ATCC

Authentication
We monitor cells to confirm that their morphology and growth properties are consistent with the originally purchased cells. If changes are noted, the cells are discarded and we return to an early passage that has been cryopreserved.

Mycoplasma contamination
HEK293T cells are mycoplasma free, and are frequently tested using the MycoAlert™ PLUS Mycoplasma Detection Kit (Lonza, cat# LT07-703)

Commonly misidentified lines (See ICLAC register)
There were no commonly misidentified cell lines

Animals and other organisms

Policy information about studies involving animals; ARRIVE guidelines recommended for reporting animal research

Laboratory animals
Drosophila melanogaster, unsexed larvae, generated in this study using attP strain M{3xP3-RFP.attP}ZH-86Fb

Mice homozygous for a “floxed” Sox2 allele were crossed with mice compound heterozygotes for a βgeo gene knocked into the Sox2 gene (generating a null Sox2 mutation), and a nestin-cre transgene(which deletes the floxed Sox2 allele specifically in the nervous system), to obtain mutant mice with homozygous Sox2 deletion in the brain (Favaro et al., 2009). Control Sox2-wild type mice are generated in the same crosses when the nestin-cre gene is not inherired, and have a Sox2 floxed allele together with an intact Sox2 gene. Mice were sacrificed at postnatal day 0 (P0) to obtain forebrains for NSC cultures (sex was indifferent). The lines (Favaro et al., 2009) are maintained by matings between cousins, and outbred every two-three generations with B6D2F1 mice, to maintain the mutant alleles.
Mice were housed at a temperature of 19-23°C, with 40-60% humidity, and a 13 hours light/11 hours dark cycle.
The experiments were approved by the Italian Ministry of Health as conforming to the relevant regulatory standards.

Wild animals
No wild animals were used in this study

Field-collected samples
No field collected samples were used in this study

Ethics oversight
Use of genetically modified animals was approved by the institutional biosafety committee. Animal ethics approval was not required for working with insects
Mice homozygous for a “floxed” Sox2 allele were crossed with mice compound heterozygotes for a βgeo gene knocked into the Sox2 gene (generating a null Sox2 mutation), and a nestin-cre transgene(which deletes the floxed Sox2 allele specifically in the nervous system), to obtain mutant mice with homozygous Sox2 deletion in the brain (Favaro et al., 2009). Control Sox2-wild type mice are generated in the same crosses when the nestin-cre gene is not inherired, and have a Sox2 floxed allele together with an intact Sox2 gene. Mice were sacrificed at postnatal day 0 (P0) to obtain forebrains for NSC cultures (sex was indifferent). The lines (Favaro et al., 2009) are maintained by matings between cousins, and outbred every two-three generations with B6D2F1 mice, to maintain the mutant alleles.
Mice were housed at a temperature of 19-23°C, with 40-60% humidity, and a 13 hours light/11 hours dark cycle.
The experiments were approved by the Italian Ministry of Health as conforming to the relevant regulatory standards.

Note that full information on the approval of the study protocol must also be provided in the manuscript.

Flow Cytometry

Plots

Confirm that:

- [x] The axis labels state the marker and fluorochrome used (e.g. CD4-FITC).
- [x] The axis scales are clearly visible. Include numbers along axes only for bottom left plot of group (a 'group' is an analysis of identical markers).
- [ ] All plots are contour plots with outliers or pseudocolor plots.
- [x] A numerical value for number of cells or percentage (with statistics) is provided.

Methodology

Sample preparation	SOX2-deleted Neural Stem Cells transduced with a GFP-SOX2-expressing lentivirus (mut+Sox2) or with the same vector (empty vector, EV) expressing GFP only for control (mut+EV) or carrying SOX2x3Mut (mut+Sox2-3x-mut), were dissociated to single cells, every 3-4 days. At every passage, aliquots of transduced cells (50,000-100,000 cells, from pooled wells) were fixed using 2% paraformaldehyde (PFA). Cells were washed twice in Phosphate Buffer Solution (PBS) and then resuspended in 200 µL of PBS to be analyzed for GFP fluorescence. A sample of non transduced SOX2-deleted Neural Stem Cells (mut nt) was treated in the same way to define the positivity threshold.
Instrument	Cytoflex S (Beckman-Coulter)
Software	CytExpert Software
Cell population abundance	Cell sorting not employed
Gating strategy	Using the FSC/SSC gating, debris wa removed by gating on the main cell population (P1 gate in blu). Positivity threshold for each samples (mut+EV, mut+Sox2, mut+Sox2-3x-mut) was defined on the basis of non transduced SOX2-deleted Neural Stem Cells (mut nt) sample. Identical positivity threshold was applied to all samples.

- [x] Tick this box to confirm that a figure exemplifying the gating strategy is provided in the Supplementary Information.
