# Supplementary material for: Structural basis for nuclear import selectivity of pioneer transcription factor SOX2
Source: Nat Commun. 2021 Jan 4;12:28. doi: 10.1038/s41467-020-20194-0 (PMC7782513; doi:10.1038/s41467-020-20194-0)
Supplement: Supplementary file 4 — Description of Additional Supplementary Files [file 41467_2020_20194_MOESM4_ESM.pdf]

Description of Additional Supplementary Files

File name: Supplementary Movie 1

Description: SOX2 conformational changes upon binding importins and nucleosomes
