# Supplementary material for: Structural basis for nuclear import selectivity of pioneer transcription factor SOX2
Source: Nat Commun. 2021 Jan 4;12:28. doi: 10.1038/s41467-020-20194-0 (PMC7782513; doi:10.1038/s41467-020-20194-0)
Supplement: Supplementary file 2 — Peer Review File [file 41467_2020_20194_MOESM2_ESM.pdf]

Reviewer #1 (Remarks to the Author):

The paper “STRUCTURAL BASIS FOR NUCLEAR IMPORT SELECTIVITY OF PIONEER TRANSCRIPTION FACTOR SOX2” by Jagga et al utilizes a wealth of biochemical, biophysical, and cellular techniques to interrogate the SOX2 nuclear import pathway, and its specificity for IMPα3. The authors highlight how the SOX2 HMG domain can impart specificity for Importin α isoforms by both regulating neighboring NLS binding and the HMG domain. The manuscript adds significantly to the current literature regarding the nuclear import of SOX2, which is a crucial tumor promoter/suppressor, and the nuclear import field. Overall, I think the paper is well written, nicely referenced, and fits well in Nature Communications. I have a few suggestions that I invite the authors to address:

Line 105-107: Unclear what this sentence is referring to. Are the authors stating the NLS1 does not bind both the major and minor NLS binding pockets at the same time or does not bind the major nor minor NLS site?
In Figure 3A, why is there a difference in SOX2 input concentrations between the R114A and all the other samples?
In Figure 4B and 4C, there are no error bars. Were these experiments performed in triplicates for statistical significance? I see there were multiple experiments performed in Figure 7F for statistical significance.
In Figure 4B and 4D, the NSC cultures transduced with SOX2x3Mut were still able to “attach to the plastic, elongate and aggregate (a possible sign of initial differentiation)” and “SOX2x3Mut may retain some activity”. What is the possible explanation for these observations that SOX2 still was able to undergo initial rounds of differentiation despite potent NLS mutations?
In Figure 6, the in vivo pull-downs show Impa1 and Impa7 does not bind to SOX2 yet does bind in the in vitro pull-downs with recombinantly expressed Importins and SOX2. Are there observations due to solely affinity differences between the Importins, or are there post-translational modifications that may be responsible?
Line 292 (page 11): “In contrast, the specificity shown by RCC1 is achieved by binding in a quite different manner involving the N-terminus of IMPα27”
I can't entirely agree with this statement, and I find much of the Discussions to be a bit off point. I interpret the authors’ findings differently. This structure provides the ultimate validation of the flexibility model proposed by Ruth et al, 2015 Structure, and later reiterated by Sankahala et al, 2017, Nat Comms. The way I read this paper, the SOX2:IMPa3 structure provides the ultimate confirmation that importin a3 structural flexibility dictates specificity for NLS-cargos. As shown by MD simulations (Pumroy et al 2015 Structure), importin a3 is the most flexible isoform able to extend and stretch in solution. The structure of importin a3 bound to RCC1 described in 2017 gave an example of a ‘stretched’ importin a3 bound a bulky cargo that clashes with the N-terminal ARMs 1-3. The structure presented in this paper illustrates a perfect example of NLS cargo that has complex interaction with the minor NLS-binding pocket. As clarified by Pumroy et al, importin a3 is flexible throughout its length. So the movement of ARM 7 to accommodate the SOX2 NLS is entirely in line with the prediction this isoform would be able to stretch in order to accommodate different substrates. This point doesn’t come across reading this paper. There’s no such thing as the ARM7 mechanism reported to bind W protein of Henipaviruses and the specificity model for RCC1. The two models are two faces of the same medal, and this paper demonstrates it. Importin a3 is just more flexible than other isoforms. It puts cargos in place by undergoing local conformational changes throughout its superhelical core. This is the main point that I believe the authors must get across in the discussions. It’s otherwise tricky for the reader to make sense of all these structures. The common denominator of all importin a3-dependent cargos is the structural complexity of the NLS that requires conformational changes to fit in the NLS-binding grove. This complexity can be caused by flanking domains that bury the NLS (e.g., RCC1 and perhaps Pb2), or a ‘trans’ bipartite NLS, as in SOX2. Therefore, I invite the authors to clearly and unambiguously discuss the flexibility of importin a3 as the key determinant for cargo-specificity.

Minor Revisions/Critiques:

Line 60: grammatical error
Line 101: grammatical error

Reviewer #2 (Remarks to the Author):

The manuscript by Jagga et al provides a detailed description of the structural basis for the physical association of SOX2 and IMPα3, which dictates entry of SOX2 into the nucleus. More specifically, this study demonstrates that the two separated nuclear localization signals (NLS) of SOX2 form a contiguous interface spanning 9 of the 10 ARM domains of IMPα3. This study also describes the structural basis for the preference of SOX2 binding to the nuclear import adapter IMPα3. Overall, this study provides new insights into the functionality of SOX2. However, this manuscript has several weaknesses, in particular how the findings of this study are presented.

1) To bolster the significance of their study, the authors argue that the two separated NLS of SOX proteins have been proposed to function independently. This is a "straw man". Although it is clear that they are separated by about 50 amino acids in the HMG domain of SOX proteins, it has been evident from published work as early as 1997 that both NLS of Sox proteins are needed for efficient nuclear entry. This has already been shown for SOX2 by Li et al, who demonstrated that mutations of either NLS only partially reduce nuclear entry and only when both NLS are mutated is virtually all nuclear entry blocked. Yes, a study published in 1997 regarding SRY and SOX9 stated in the title of the paper that the two NLS of SRY and SOX9 work independently. However, no data that they work separately was presented in that study. What was shown is that disruption of either the N-terminal bipartite NLS or the C-terminal NLS reduced nuclear localization. Separated does not mean independent. Consequently, it would be more appropriate for the authors to argue that previous work did not establish how the two separated NLSs work together to promote efficient nuclear entry. The data presented in their study provides an elegant answer to that question. As a case in point, on line 263, where the authors use the word "independent", it would be clearer if they substituted the work "separated"

2) This study uses crystal structures to show that two NLS of SOX2 form a contiguous interface spanning 9 of the 10 ARM domains of IMPα3. This study also shows that mutating either the N-terminal NLS or the C-terminal NSL of recombinant SOX2 drastically reduces binding to recombinant IMPα3. Additionally, this study shows that the 3x mutant, which mutates both NLS, drastically blocks nuclear entry. However, they do not show that mutating either of the NLS mutants on their own is sufficient to block nuclear entry. Consequently, the authors should refer to the work of Li et al and point out that these workers demonstrated that both NLS need to be disrupted to dramatically reduce nuclear entry, whereas mutating one or the other NLS only partially reduces nuclear entry.

3) The findings reported in this study for SOX2 provide a model for understanding the nuclear entry of SOX proteins more generally. The authors could have provided additional data to support this point by generating co-crystals of another SOX protein (e.g. SRY or SOX9) with IMPα3. At the very least, the authors should discuss in more detail published studies conducted other SOX proteins that are consistent with their findings for SOX2. For example, specific SRY mutants, such asR75G,R76L,K77T mutant of SRY (part of the conserved N-terminal partite NLS) and similar mutants of SOX9, which reduce nuclear entry. In doing so, the authors should explain why their R57A mutant did not reduce binding to recombinant IMPα3. This would provide an opportunity for the authors to point out that the association of SOX2 with IMPα3 is only part of the story for nuclear import. For other SOX proteins, calmodulin has also been show to play an important role in nuclear entry.

Minor issues

4) The authors should point out that no naturally occurring mutants in the NLS of SOX2 have been described.

5) The two NLS of SOX2 proteins are considered part of the highly conserved HMG domain found in all SOX proteins. The two NLS do not flank the HMG domain as described in this manuscript.

6) The authors should point out in the text of the results section that their co-crystal between SOX2 and IMPα3 was made using the HMG domain of SOX2 and a few amino acids flanking the C-terminus of the HMG domain (residues 39–127). As the authors know, but the reader may not, full length SOX2 does not form crystals, due to unstructured regions of the molecule.

7) Supplementary Figure 1 should be omitted. Reference 32 of their paper discusses the roles of each SOX protein in multiple tissues.

8) The data showing that ectopic expression of wild-type Dichaete (SOX2 homologue in Drosophila), but not mutant Dichaete, was not provided. Either show the data or state “data not shown”. If they can, it is recommended that they show the data, even as a supplemental figure.

9) The authors should explain what is meant by Mut1 and Mut2, which were used in Figure 4BC and Supplemental Figure 2.

10) Line 46 of the Abstract states ….. two distantly positioned NLSs …. Should be changed to ….. two distantly positioned NLSs of SOX2 …..

Nature Communications NCOMMS-20-26952A

RESPONSE TO REVIEWERS

Reviewers' comments are in Italic and responses are in red Roman typeface.

Reviewer #1 (Remarks to the Author):

The paper "STRUCTURAL BASIS FOR NUCLEAR IMPORT SELECTIVITY OF PIONEER TRANSCRIPTION FACTOR SOX2" by Jagga et al utilizes a wealth of biochemical, biophysical, and cellular techniques to interrogate the SOX2 nuclear import pathway, and its specificity for IMPα3. The authors highlight how the SOX2 HMG domain can impart specificity for Importin α isoforms by both regulating neighboring NLS binding and the HMG domain. The manuscript adds significantly to the current literature regarding the nuclear import of SOX2, which is a crucial tumor promoter/suppressor, and the nuclear import field. Overall, I think the paper is well written, nicely referenced, and fits well in Nature Communications. I have a few suggestions that I invite the authors to address:

RESPONSE: We thank the reviewer for their positive comments regarding our manuscript

Line 105-107: Unclear what this sentence is referring to. Are the authors stating the NLS1 does not bind both the major and minor NLS binding pockets at the same time or does not bind the major nor minor NLS site?

RESPONSE: We have reworked this part of the manuscript to clarify this point. The text now reads:

Lines 103-109: "SOX2 bound IMPα3 through an extensive and contiguous interface across ARM domains 1-9 of IMPα3 (**Figure 2**). The N-terminal NLS (NLS1) was previously reported to be bipartite[5,13,31], and therefore expected to be bound at both the major and minor sites on IMPα3. However, we found instead that, SOX2 residues Arg40, Lys42, and Arg43 were bound at the minor site (IMPα3 ARM domains 6-8; **Figure 2**) and that SOX2 Arg57 was bound at ARM 9, outside of the minor site."

In Figure 3A, why is there a difference in SOX2 input concentrations between the R114A and all the other samples?

RESPONSE: The levels of SOX2 proteins used were in excess to the bead binding capacity. Therefore, although the input was slightly lower in this one sample, the amounts of SOX proteins immobilised on the beads were equivalent, as was demonstrated in the pull-down.

In Figure 4B and 4C, there are no error bars. Were these experiments performed in triplicates for statistical significance? I see there were multiple experiments performed in Figure 7F for statistical significance.

RESPONSE: The growth curves for mutant NSC transduced with wild type Sox2, mutant NSC transduced with empty vector, and mutant NSC transduced with SOX2x3Mut, were performed separately with mutant cultures from two different mutant brains; these are reported, separately, in Fig. 4 and in Suppl. Fig. 2. Here we show (for the Referee only), the overlap between the two experiments. As the interval between consecutive passages is about 3.5 days, each experiment lasts almost two months. Each passage requires the pooling of nine wells for each mutant, a careful and thorough but gentle dissociation, counting the cells, and replating. This takes a substantial length of time and, in practice, we could not perform the two experiments in parallel to avoid delays that would compromise the quality of the cells. For these reasons, the second experiment was performed after the end of the first one. Having two experiments, we did not report error bars. However, it is quite clear from the overlap of the two figures that the growth curves of the two different mutants are quite well superimposable for each of the tested conditions, and the differences between the three types of NSC (transduced with Sox2, transduced with EV, transduced with SOX2x3Mut) are always evident along the whole extent of the growth curve.

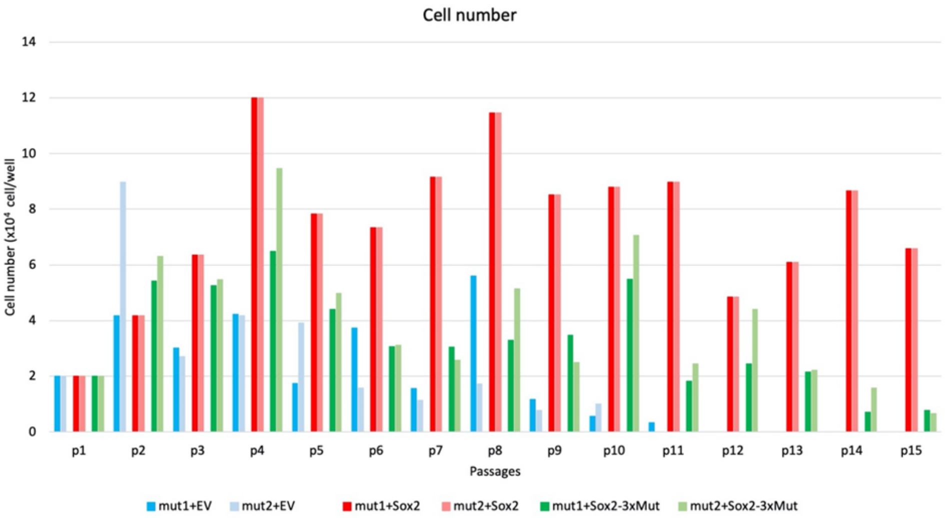

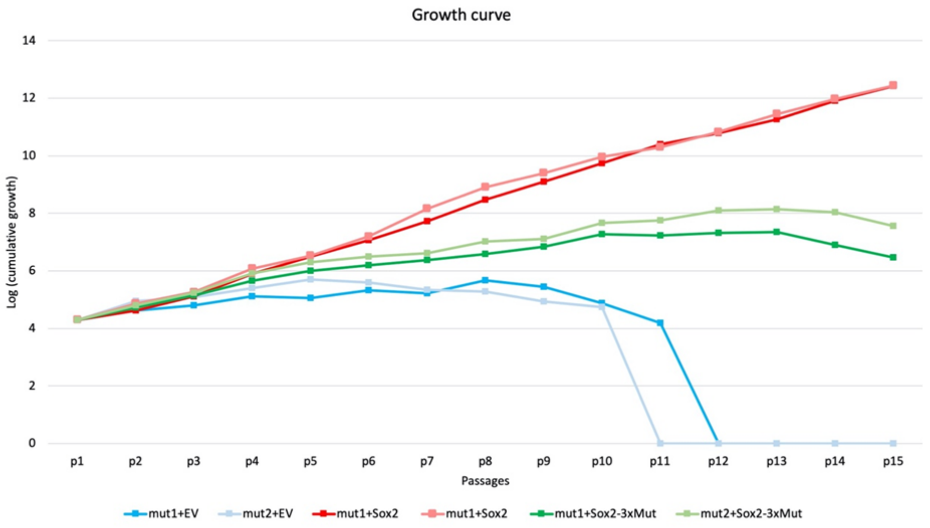

In Figure 4B and 4D, the NSC cultures transduced with SOX2x3Mut were still able to "attach to the plastic, elongate and aggregate (a possible sign of initial differentiation)" and "SOX2x3Mut may retain some activity". What is the possible explanation for these observations that SOX2 still was able to undergo initial rounds of differentiation despite potent NLS mutations?

RESPONSE: As shown by confocal microscopy, a small amount of SOX2x3Mut still finds its way into the nucleus. Considering that SOX2 is overexpressed, it is possible that this is enough to sustain some degree of proliferation, as shown by the growth curve, and of differentiation. The abnormal morphology of the cells transduced with Sox2x3Mut will be the subject of a future investigation; we speculate that cytoplasmic SOX2 might retain in the cytoplasm other transcription factors (among SOX2 interactors), altering the nuclear balance of transcriptional factors required for proper cell functioning.

In Figure 6, the in vivo pull-downs show Impa1 and Impa7 does not bind to SOX2 yet does bind in the in vitro pull-downs with recombinantly expressed Importins and SOX2. Are there observations due to solely affinity differences between the Importins, or are there post-translational modifications that may be responsible?

RESPONSE: It is not that unusual for weaker interacting proteins to be detected in an *in vitro* system using two pure proteins, whilst in the context of a cell (with many competing factors), the same interaction can't be detected. Overall, we believe that these complementary systems are showing a consistent pattern of binding, with IMPA3 showing high affinity interaction with SOX2, while the other IMPA family members exhibiting a weaker interaction. This is also consistent with the interfaces observed through structural approaches.

Line 292 (page 11): "In contrast, the specificity shown by RCC1 is achieved by binding in a quite different manner involving the N-terminus of IMPα27"
I can't entirely agree with this statement, and I find much of the Discussions to be a bit off point. I interpret the authors' findings differently. This structure provides the ultimate validation of the flexibility model proposed by Ruth et al, 2015 Structure, and later reiterated by Sankahala et al, 2017, Nat Comms. The way I read this paper, the SOX2:IMPa3 structure provides the ultimate confirmation that importin a3 structural flexibility dictates specificity for NLS-cargos. As shown by MD simulations (Pumroy et al 2015 Structure), importin a3 is the most flexible isoform able to extend and stretch in solution. The structure of importin a3 bound to RCC1 described in 2017 gave an example of a 'stretched' importin a3 bound a bulky cargo that clashes with the N-terminal ARMs 1-3. The structure presented in this paper illustrates a perfect example of NLS cargo that has complex interaction with the minor NLS-binding pocket. As clarified by Pumroy et al, importin a3 is flexible throughout its length. So the movement of ARM 7 to accommodate the SOX2 NLS is entirely in line with the prediction this isoform would be able to stretch in order to accommodate different substrates. This point doesn't come across reading this paper. There's no such thing as the ARM7 mechanism reported to bind W protein of Henipaviruses and the specificity model for RCC1. The two models are two faces of the same medal, and this paper demonstrates it. Importin a3 is just more flexible than other isoforms. It

puts cargos in place by undergoing local conformational changes throughout its superhelical core. This is the main point that I believe the authors must get across in the discussions. It's otherwise tricky for the reader to make sense of all these structures. The common denominator of all importin a3-dependent cargos is the structural complexity of the NLS that requires conformational changes to fit in the NLS-binding grove. This complexity can be caused by flanking domains that bury the NLS (e.g., RCC1 and perhaps Pb2), or a 'trans' bipartite NLS, as in SOX2. Therefore, I invite the authors to clearly and unambiguously discuss the flexibility of importin a3 as the key determinant for cargo-specificity.

RESPONSE: We have modified the text to address this point more explicitly. We have removed the text "In contrast, the specificity shown by RCC1 is achieved by binding in a quite different manner involving the N-terminus of IMPα27". We have also incorporated a discussion of IMPA3 flexibility. The text now reads:

Lines 309-314: The greater flexibility of IMPα3 compared with other IMPα isoforms is important for its binding RCC1 selectively[27,49], whereas we have shown here that the differential positioning of ARM7 in IMPα3 (the position of which does not change relative to ARM6 and ARM8 in all published IMPα3 structures – see Supplementary Figure 5) makes an important contribution to its selective binding of SOX2, similar to that seen for the W protein of Henipaviruses[50] (**Figure 8**).

Minor Revisions/Critiques:
Line 60: grammatical error
Line 101: grammatical error

RESPONSE: These have been corrected.

Reviewer #2 (Remarks to the Author):

The manuscript by Jagga et al provides a detailed description of the structural basis for the physical association of SOX2 and IMPα3, which dictates entry of SOX2 into the nucleus. More specifically, this study demonstrates that the two separated nuclear localization signals (NLS) of SOX2 form a contiguous interface spanning 9 of the 10 ARM domains of IMPα3. This study also describes the structural basis for the preference of SOX2 binding to the nuclear import adapter IMPα3. Overall, this study provides new insights into the functionality of SOX2. However, this manuscript has several weaknesses, in particular how the findings of this study are presented.

1) To bolster the significance of their study, the authors argue that the two separated NLS of SOX proteins have been proposed to function independently. This is a "straw man". Although it is clear that they are separated by about 50 amino acids in the HMG domain of SOX proteins, it has been evident from published work as early as 1997 that both NLS of Sox proteins are needed for efficient nuclear entry. This has already been shown for SOX2 by Li et al, who demonstrated that mutations of either NLS only partially reduce nuclear entry and only when both NLS are mutated is virtually all nuclear entry blocked. Yes, a study published in 1997 regarding SRY and SOX9 stated in the title of the paper that the two NLS of SRY and SOX9 work

independently. However, no data that they work separately was presented in that study. What was shown is that disruption of either the N-terminal bipartite NLS or the C-terminal NLS reduced nuclear localization. Separated does not mean independent. Consequently, it would be more appropriate for the authors to argue that previous work did not establish how the two separated NLSs work together to promote efficient nuclear entry. The data presented in their study provides an elegant answer to that question. As a case in point, on line 263, where the authors use the word "independent", it would be clearer if they substituted the work "separated"

RESPONSE: We have removed "independent". How these two NLSs, distally positioned at either end of a HMG domain form a continuous binding interface on IMPA is the major focus of our work.

2) This study uses crystal structures to show that two NLS of SOX2 form a contiguous interface spanning 9 of the 10 ARM domains of IMPα3. This study also shows that mutating either the N-terminal NLS or the C-terminal NSL of recombinant SOX2 drastically reduces binding to recombinant IMPα3. Additionally, this study shows that the 3x mutant, which mutates both NLS, drastically blocks nuclear entry. However, they do not show that mutating either of the NLS mutants on their own is sufficient to block nuclear entry. Consequently, the authors should refer to the work of Li et al and point out that these workers demonstrated that both NLS need to be disrupted to dramatically reduce nuclear entry, whereas mutating one or the other NLS only partially reduces nuclear entry.

RESPONSE: We agree with the reviewer, and thank them for pointing this out. We have now incorporated reference to the Li et al study into the manuscript:

Lines 131-135: "Based on a previous study demonstrating that mutating both SOX2 NLS regions impart the most dramatic reduction in nuclear localisation[33] and combining our knowledge of the structural interface, we designed a SOX2 K42A, R43A, and K115A triple mutant (SOX2x3Mut) and tested the effect on the cell biology processes that SOX2 mediates".

3) The findings reported in this study for SOX2 provide a model for understanding the nuclear entry of SOX proteins more generally. The authors could have provided additional data to support this point by generating co-crystals of another SOX protein (e.g. SRY or SOX9) with IMPα3. At the very least, the authors should discuss in more detail published studies conducted other SOX proteins that are consistent with their findings for SOX2. For example, specific SRY mutants, such as R75G,R76L,K77T mutant of SRY (part of the conserved N-terminal partite NLS) and similar mutants of SOX9, which reduce nuclear entry. In doing so, the authors should explain why their R57A mutant did not reduce binding to recombinant IMPα3. This would provide an opportunity for the authors to point out that the association of SOX2 with IMPα3 is only part of the story for nuclear import. For other SOX proteins, calmodulin has also been show to play an important role in nuclear entry.

RESPONSE: We agree with the reviewer that these structures provide a model for understanding of SOX proteins more generally. We are in the process of attempting to crystallise other SOX protein family members, however this is a very lengthy and

demanding process owing to the flexibility of the importins. As requested by the reviewer, we have included additional text to compare published studies conducted of other SOX proteins, including specific SRY mutants. Additionally, we have also provided an explanation as to why our R57A mutant did not reduce binding to recombinant IMPα3, and provided additional discussion around other regulatory mechanisms such as calmodulin. The incorporated text is as follows:

Lines 276-290: The structural insights from our study may also assist with contextualising how mutations in other SOX proteins may cause aberrations in nuclear transport and disease. Whilst there are no naturally occurring mutants in the NLSs of SOX2 that have been documented, SRY mutants have been shown to impede nuclear localisation and result in sex reversal. Mutations such as SRY R62G[13], R75M[48], and R76P[49], located within the NLS1 (bipartite region) of SOX proteins, were shown to bind within the IMPA minor site (IMPα3 ARMs 6-8) and ARM 9 in this study. Similarly, the NLS2 region harbours mutations such as SRY R133W[50], shown to bind at the major site of IMPα3 (within ARM3). It is unlikely however that the interfaces identified in this study can be used to attribute all disease causing mutations across the SOX family since these sites are also subject to complex regulation including calmodulin binding (also shown to regulate nuclear import. This may explain for example why some disease causing mutations, such as SRY R76P[49] (equivalent to SOX2 Arg57), shown to important for nuclear import regulation through calmodulin, did not disrupt the IMPA3:SOX2 interaction[6].

Minor issues
4) The authors should point out that no naturally occurring mutants in the NLS of SOX2 have been described.

RESPONSE: We have incorporated this into the text on line 278.

5) The two NLS of SOX2 proteins are considered part of the highly conserved HMG domain found in all SOX proteins. The two NLS do not flank the HMG domain as described in this manuscript.

RESPONSE: We agree with the reviewer. We have now made this point explicitly and modified the text as follows:

Line 500: The SOX family is comprised of 20 members, each containing a highly conserved HMG-domain (in green/purple), with NLSs positioned within the extremities of the HMG-domain.

6) The authors should point out in the text of the results section that their co-crystal between SOX2 and IMPα3 was made using the HMG domain of SOX2 and a few amino acids flanking the C-terminus of the HMG domain (residues 39–127). As the authors know, but the reader may not, full length SOX2 does not form crystals, due to unstructured regions of the molecule.

RESPONSE: The domain constructs were presented in the Materials and Methods, however we have also added this information to the results section. The modified text now reads:

Lines 97-100: To better understand the mechanisms of how these critical signaling regions in SOX proteins interact with nuclear import receptors to drive nuclear transport, we crystallized the HMG-domain of SOX2 (comprising residues 39-127) in complex with different IMPα isoforms.

7) Supplementary Figure 1 should be omitted. Reference 32 of their paper discusses the roles of each SOX protein in multiple tissues.

REPONSE: This has been removed, and figures have been renumbered accordingly.

8) The data showing that ectopic expression of wild-type Dichaete (SOX2 homologue in Drosophila), but not mutant Dichaete, was not provided. Either show the data or state "data not shown". If they can, it is recommended that they show the data, even as a supplemental figure.

RESPONSE: We have incorporated that additional data as requested and this has been included as a supplementary figure. The modified text reads:

Lines 181-187: Ectopic expression of Dichaete results in developmental defects when expressed from a variety of promoters[36-38], and we also observed that no ptc-Gal4, UAS-Dichaete adults emerged (0/63 siblings, **Supplementary Figure 3**) indicating that it results in lethality when raised at 25□C). In contrast, expression of Dichaete3xMut had no effects upon development and *ptc-Gal4, UAS-Dichaete3xMut* animals emerged at approximately a Mendelian ratio (37/93 siblings, **Supplementary Figure 3**).

9) The authors should explain what is meant by Mut1 and Mut2, which were used in Figure 4BC and Supplemental Figure 2.

RESPONSE: We have modified and incorporated the text to make this clearer. It now reads:

Lines 146-154*: Sox2*$^{-/-}$ NSC transduced with wild type SOX2 recovered the ability to efficiently self-renew (**Figure 4B,C**), growing with kinetics comparable to wild-type NSC (not shown). In contrast, NSC transduced with the SOX2x3Mut demonstrated inefficient expansion, progressively slowing until a plateau was reached, after which their numbers started to decline (**Figure 4B,C**). In an independent experiment with NSC from a different Sox2 mutant mouse transduced with the same vectors, essentially identical results were obtained, with growth curves closely overlapping those of the first experiment (**Supplementary Fig. 1**).

10) Line 46 of the Abstract states ….. two distantly positioned NLSs …. Should be changed to ….. two distantly positioned NLSs of SOX2 …..

RESPONSE: We have corrected this. The text now reads:

Lines 45-47: Unexpectedly, we find here that these two distantly positioned NLSs of SOX2 contribute to a contiguous interface spanning 9 of the 10 ARM domains on the

nuclear import adapter IMPα3.

Reviewer #1 (Remarks to the Author):

I am satisfied by the way the authors revised the paper in response to my criticisms. I think this work is now ready to be disseminated to the scientific community.

Reviewer #2 (Remarks to the Author):

The authors have addressed the concerns raised by this reviewer. There is one minor issue. On line 288, a word has been omitted. Did the authors mean to write ...shown to "be" important...

Nature Communications NCOMMS-20-26952A

RESPONSE TO REVIEWERS

Reviewers' comments are in Italic and responses are in red Roman typeface.

Reviewer #1 (Remarks to the Author):

I am satisfied by the way the authors revised the paper in response to my criticisms. I think this work is now ready to be disseminated to the scientific community.

Reviewer #2 (Remarks to the Author):

The authors have addressed the concerns raised by this reviewer. There is one minor issue. On line 288, a word has been omitted. Did the authors mean to write ...shown to "be" important...

RESPONSE: We thank the reviewer. This has been corrected in final submitted version
